# Clustering suicidal phenotypes and genetic associations with brain-derived neurotrophic factor in patients with substance use disorders

**DOI:** 10.1038/s41398-021-01200-5

**Published:** 2021-01-21

**Authors:** Romain Icick, Vanessa Bloch, Nathalie Prince, Emily Karsinti, Jean-Pierre Lépine, Jean-Louis Laplanche, Stéphane Mouly, Cynthia Marie-Claire, Georges Brousse, Frank Bellivier, Florence Vorspan

**Affiliations:** 1 Assistance Publique-Hôpitaux de Paris (AP-HP), Groupe Hospitalier Saint-Louis–Lariboisière–Fernand Widal, Paris, France; 2 INSERM U1144, “Therapeutic Optimization in Neuropsychopharmacology”, Paris, France; 3 Université de Paris, Inserm UMR-S1144, Paris, France; 4 ED139, Paris Nanterre University, Nanterre, France; 5 Inserm UMR-1107, Neuro-Dol, Université Clermont-Auvergne, Clermont-Ferrand, France

**Keywords:** Addiction, Clinical genetics

## Abstract

Suicide attempts (SA), especially recurrent SA or serious SA, are common in substance use disorders (SUD). However, the genetic component of SA in SUD samples remains unclear. Brain-derived neurotrophic factor (BDNF) alleles and levels have been repeatedly involved in stress-related psychopathology. This investigation uses a within-cases study of BDNF and associated factors in three suicidal phenotypes (‘any’, ‘recurrent’, and ‘serious’) of outpatients seeking treatment for opiate and/or cocaine use disorder. Phenotypic characterization was ascertained using a semi-structured interview. After thorough quality control, 98 SNPs of BDNF and associated factors (the BDNF pathway) were extracted from whole-genome data, leaving 411 patients of Caucasian ancestry, who had reliable data regarding their SA history. Binary and multinomial regression with the three suicidal phenotypes were further performed to adjust for possible confounders, along with hierarchical clustering and compared to controls (*N* = 2504). Bayesian analyses were conducted to detect pleiotropy across the suicidal phenotypes. Among 154 (37%) ever suicide attempters, 104 (68%) reported at least one serious SA and 96 (57%) two SA or more. The median number of non-tobacco SUDs was three. The BDNF gene remained associated with lifetime SA in SNP-based (rs7934165, rs10835210) and gene-based tests within the clinical sample. rs10835210 clustered with serious SA. Bayesian analysis identified genetic correlation between ‘any’ and ‘serious’ SA regarding rs7934165. Despite limitations, ‘serious’ SA was shown to share both clinical and genetic risk factors of SA—not otherwise specified, suggesting a shared BDNF-related pathophysiology of SA in this population with multiple SUDs.

## Introduction

The burden of suicidal behavior is particularly high among individuals suffering from substance use disorders (SUD). About one in three individuals seeking treatment in specialized care centers for SUD report at least one lifetime suicide attempt (SA)^1,2^ and up to 35% deaths in heroin-dependent subjects have been attributed to suicide^3^. This may arise from people with SUD reporting more ‘recurrent’ and/or ‘serious’ SA^1,4,5^. A focus on risk factors associated with such specific patterns of lifetime SA should help prevent suicide deaths in these high-risk populations.

According to the “stress-diathesis” model, suicidal behavior is a multifactorial phenomenon that relies on both individual vulnerability and precipitating risk factors, including temporally distal and/or proximal events^6^. The biological underpinnings to suicidal behavior vulnerability, the diathesis, seems at least partly independent from other psychiatric disorders^7,8^. Genome-wide association studies were initially inconclusive regarding SA in major depressive, bipolar and schizophrenic disorders^9^. However, the use of a severity-based SA phenotype showed significant associations, including: one single nucleotide polymorphism (SNP) with the severity of the worst SA lifetime in psychiatric populations^10^; and three SNPs in a general population cohort reporting deliberate self-harm ranging from “no idea nor attempt” to “suicide attempt”^11^. However, no genome-wide significant association has been found in suicide decedents^12^. Most of the significant loci in these studies were mapped to genes involved in metabolic/inflammatory pathways.

Other promising candidate pathways for suicidal behavior have also been identified, including the serotonergic, hypothalamic-pituitary-adrenal (HPA) axis and the neurotrophin systems^13^. These pathways require investigation in SUD populations. Population samples with a high load of non-genetic SA risk factors (including early and middle-life stressors, impulsivity, comorbid substance use and psychiatric disorders), would be expected to show differences in SA prevalence that may be explained by genetic factors. Given extant knowledge regarding how SUDs and SAs occur (and co-occur) across the lifespan, those genetic factors can be expected to be found in biological pathways that play a key role in neuroadaptation to stressors. For instance, in cocaine dependence, two studies from the same research group reported significant associations between any SA and variants in the HPA axis gene *FKBP5*, both alone^14^ and with *CRHBP*^15^. In alcohol dependence, evidence was inconclusive regarding associations of SA with the *COMT* gene^16^, dopaminergic receptor genes^17,18^, the serotoninergic system through *TPH2* polymorphisms^19^ and the glutamatergic gene *GRIN2B*^20^. Notably, these studies relied on a low number (1–5) of genetic variants, thereby increasing the risk of both false negative and false positive findings. Finally, a recent whole-genome transcriptomics study has reported 222 differentially expressed genes between the presence vs. absence of lifetime SUD in suicide decedents^21^, opening new avenues for prioritized candidate gene studies.

Brain-derived neurotrophic factor (BDNF), and associated inducers and neurotrophins (the ‘neurotrophic pathway’) is a promising candidate, being involved in the neurobiological processes of stress reactivity^22^, brain plasticity in response to chronic drug use^23^ and suicidal behavior. Neurotrophins are growth factors involved in the regulation of cerebral plasticity, notably synaptic changes in structure, number and connectivity. BDNF is the most studied and the most widely distributed neurotrophin. BDNF is one of the most replicated pathways in genetic studies on SA^13^. However, plasma or cerebrospinal fluid BDNF levels do not seem to be associated with SA risk^24^, so that associations between the BDNF and the ‘neurotrophic pathway’ and SA may involve gene variants with complex or indirect impacts on gene expression or proteins, most likely during time-limited specific stress-response.

Based on these observations, we undertook a study of SA and ‘neurotrophic pathway’ genes in a large sample of outpatients seeking treatment for SUD, who were extensively characterized for their genotypes and both their suicidal and substance use phenotypes. We hypothesized that at least one SNP in the ‘neurotrophic pathway’ would be associated with any, serious or recurrent SA (or a combination of these phenotypes).

## Methods

This was a hypothesis-driven, candidate-pathway genetic association study using a within-cases design followed by a case-control design. It was conducted in a clinical sample that was previously analyzed in part regarding clinical aspects of SA^1,4^. Of note, throughout this manuscript, the term substance use disorders (SUDs) will refer to substance abuse or dependence according to the Diagnostic and Statistical Manual of mental disorders, 4th edition—text revised (DSM-IV-TR) classification^25^.

### Sample recruitment

Treatment-seeking outpatients attending tertiary care programs in the Paris area were recruited through two multicentre research protocols. Participants were French-speaking, 18+ years old individuals, seeking treatment in any of the participating centers. Further inclusion criteria were:

- protocol one (seven sites, 2008–2012) = receiving stable methadone treatment for three months or more for treating opioid dependence [see ref. ^26^];
- protocol two (nine sites, 2012–2016) = any past-year cocaine use [see ref. ^27^].

For this study, participants had to fulfill criteria for either lifetime opiate or cocaine abuse/dependence according to DSM-IV-TR^25^.

Patients were excluded if they were undergoing compulsory treatment or were unable to consent for any other reason (non-French speaking, major cognitive impairment).

Both protocols were approved by the local ethics committees (CPP Ile-de-France VI for study one and CPP Ile de France IV for study two) and preregistered (clinicaltrials.gov NCT00894452 and NCT01569347, respectively), and by the relevant Institutional Review Board for further analyses of the combined sample [CEEI from the *Institut de la Santé et de la Recherche Médicale* (INSERM), IRB00003888 in July 2015]. All participants provided written informed consent, and study records were continuously monitored by the local research administration (Unité de Recherche Clinique) to ensure their conformity to the original protocols. The authors assert that all procedures contributing to this work comply with the ethical standards of the relevant national and institutional committees on human experimentation and with the Helsinki Declaration of 1975, as revised in 2008.

### Clinical assessments

Data were obtained during a single interview conducted by trained psychologists or M.D.s.

- Sociodemographic conditions were collected using a standard procedure, and included the number of years in education starting from elementary school, dichotomized employment and marital statuses, as well as history of homelessness, which was retained if the participant reported having spent at least three months living on the streets. We chose this stringent definition^28^ since several recruitment centers dealt with particularly low-income populations, among whom brief or sheltered homelessness is common;
- Past history of SA was characterized using the ‘suicide’ section of the Diagnostic Interview for Genetic Studies, v 4.0 (DIGS 4.0)^29^, where the screening question refers to “kill oneself”, followed by the number of times the respondent endorses this affirmation then an assessment of the self-reported “worst attempt”. Taken altogether, this multiple-items assessment of SA history enhances data reliability, as previously reported in general population samples^30^. This is of further relevance in SUD populations, where drug overdoses (which were assessed specifically in the study, data not shown) can be mistakenly considered as accidental^31^. Along with the presence and the number of lifetime SA, the questionnaire also focused on what participants regarded as their worst SA ever, which was dichotomized into self-poisoning or violent means (jumping, hanging, shooting). For this particular SA, the degree of intentionality and age of occurrence were noted and the medical treatment that followed was also collected. Besides the presence of lifetime (‘any’) SA, two additional phenotypes were considered:
  - *Serious SA* was deemed present if the respondent’s worst SA was either made with a violent method, or if a medical treatment was necessary (regardless of the method that was used), as previously defined^4,32^. This definition thus refers to either the actual or the potential lethality of an attempt;
  - *Recurrent SA is* defined as reporting two or more attempts over the lifespan. This cutoff has previously been used for study outcomes of first suicide attempts^33,34^.
  In the sample, 114 patients (64% of ever attempters) reported ‘serious’ SA and 102 (62% of ever attempters) reported at least 2 lifetime, ‘any’ SA;
- Lifetime patterns of use were characterized for each substance through the E section of the Structured Clinical Interview for DSM-IV (SCID-IV)^35^, comprising diagnoses of SUD and age at onset (AAO) of substance use and of SUD. Current tobacco smoking was further assessed by measuring nicotine dependence on the Heaviness of Smoking Index (HSI)^36^, a validated measure combining the delay to first smoke after wake-up and the number of cigarettes smoked per day (CPD). A cut-off score ≥4 was shown to reliably identify nicotine-dependent smokers^37^. The variable ‘PolySUD’ was defined as having a number of lifetime DSM-IV SUD above three, which was the sample’s median value.
- History of treatment with methadone or buprenorphine (approved treatments for opioid addiction in France) in all forms were also collected^38^;
- Ongoing treatment for mood disorders^39,40^ was also collected and used as an empirical proxy for identifying current/recent mood disorders.
- Psychiatric hospitalizations, which was coded present when participants reported at least one psychiatric hospitalization for any psychiatric indication;

### General population control sample

2,504 individuals of Caucasian ancestry genotyped at the Wellcome Trust Institute (UK Blood Service Control Group genotyped using the Affymetrix v6.0 chip, dataset number EGAD00010000290, downloaded March 2019) were considered as a control sample to further assess population stratification, as in previous psychiatric genetics studies^41^. The UK general population was deemed comparable to the French population regarding daily tobacco smoking (16–20% vs. 20–24%)^42^, use of other substances (alcohol, stimulants, opioids among respondents aged 15–34)^43^ and importantly, prevalence of lifetime SA, which was 7% according to both countries’ most recent surveys^44,45^. Unadjusted suicide deaths rates per 100,000, however, were higher in France than in UK in 2016^46^. Control subjects were all of Caucasian ancestry.

### Biological sampling and genotyping

Patients’ DNA was extracted from whole blood using a Maxwell 16 PROMEGA® extractor (Promega France, Charbonnières-les-Bains, France). Purity assessment followed the procedures described by the *Centre National de Recherche en Génomique Humaine*, estimated on a NanoDrop® spectrophotometer. Participants were genotyped using the Infinium PsychArray (https://www.illumina.com/products/by-type/microarray-kits/infinium-psycharray.html). Samples were processed in two stages (2014 and 2017) by Integragen SA® (Evry, France) and genotype files were merged for this study, keeping variants common to both extractions.

Controls’ DNA was extracted and analyzed according to the Wellcome Trust Case-Control Consortium standards. Genotyping was performed with the HumanHap550 array (Illumina, San Diego, CA, USA; https://support.illumina.com/array/array_kits/humanhap550-quad_plus_dna_analysis_kit.html).

### Gene selection, SNP selection, and genome-wide quality control

**Fig. 1:**
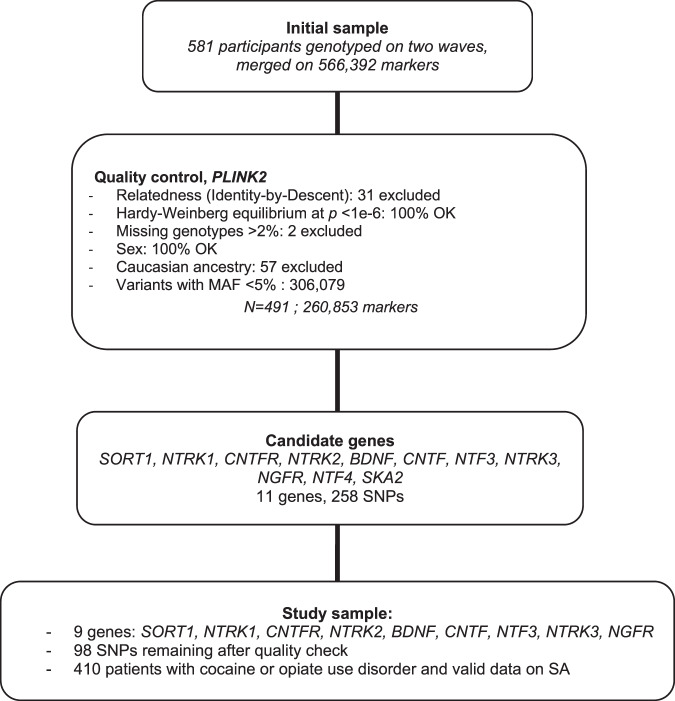
Study flowchart for sample and SNP selection. MAF, minor allele frequency; SNP, single nucleotide polymorphism; SA, suicide attempt.

We applied, a priori, a custom selection of autosomal genes from the three main neurotrophin signaling pathways identified through the *Online Mendelian Inheritance in Man* (OMIM) database. We limited the selection to extracellular ligands/receptors, so as to avoid gathering too many unspecific genes from intracellular effectors. We included genes from (i) the ligands *BDNF*, *NTF3, NTF4, NGFB, NGFA, CNTF* and their receptors *NTRK2, NTRK3*, and *CNTFR*, (ii) the major regulator of BDNF function sortilin (*SORT1*) and (iii) *SKA2*, a gene involved in stress reactivity, recently and repeatedly associated with SAs^47,48^. Those eleven genes harbored 258 markers in our DNA array (see Fig. 1). HG19 was the reference human genome version. The full list of variant names, their correspondence with Illumina® positions, and gene length and coverage by the DNA array are listed in Supplementary Table 1.

PLINK software^49^ was used for quality control, following on a consensus procedure^50^ (see flowchart, Fig. 1). At the whole-sample (*N* = 581) and genome-wide (566,932 SNPs) levels, two individuals with >2% genotype missingness were excluded. There was no sex discrepancy, no deviation from Hardy-Weinberg Equilibrium at *p* < 1e−6, or markers with more than 2% missing genotypes. We considered only SNPs*,* i.e. markers with minor allele frequency (MAF) ≥ 5%, for analysis. This left 579 individuals and 260,853 markers. Then, cryptic relatedness by Identity-by-Descent was searched for, using a pi-hat threshold of 0.2 (2nd degree relatedness), and Caucasian ancestry was assessed according to genetic proximity with known Caucasian populations described in the 1000 genomes project (1000G) panel, based on visual inspection of principal component analysis (*N* = 2,502 from five superpopulations, plot available from the authors by request). As a result, 31 individuals with the lowest genotyping rates from 62 pairs (58 pairs were duplicate inclusions between original protocols, two participants were known brothers and two were unknown cousins or amplification mismatch according to piHat scores) and another 57 due to non-Caucasian ancestry were excluded. There were 411 patients with either opioid or cocaine use disorder and valid data regarding lifetime SA in the remaining 491 individuals. Ninety-eight SNPs from 9 genes of the ‘neurotrophic pathway’ remained after quality control (*SORT1, NTRK1, CNTFR, NTRK2, BDNF, CNTF, NTF3, NTRK3, NGFR*; Fig. 1, markers with *; supplementary Table 1). Control samples underwent the same quality control pipeline, leaving 2,502 individuals with 119,171 variants shared between the two genotyping methods, for merging with the cases’ sample (see below).

Variants showing significant associations with any of those phenotypes were annotated for their biological function and plausible impact according to multiple online databases, as previously suggested^51^: (1) the Combined Annotation Dependent Depletion (CADD) database, where a scaled (‘Phred’) score ≥10 indicates that a given variant belongs to the 10% most deleterious substitutions of the human genome^52^, (2) brain expression and methylation quantitative trait loci (eQTL and mQTL) provided by GTEx portal^53^ and mQTLdb (middle-age data^54^) databases, respectively, and (3) the RegulomeDB database for non-exonic variants, which assesses their propensity to bind DNA enzymes and/or modify DNA conformation^55^.

### Statistical analyses

Data are expressed by mean ± standard deviation (SD) or median and interquartile range (IQR) for continuous variables and counts and frequencies for nominal variables, as appropriate. For the sake of brevity, only descriptive values of the main clinical and sociodemographic variables are reported. These variables were tested one-by-one in bivariate tests with suicidal phenotypes. For ‘any’ lifetime SA, SNPs were tested under the genotypic, trend, allelic, recessive and dominant models, using PLINK—*model* specifier, gene-based tests using PLINK—*set-test* function. For ‘serious’ and ‘recurrent’ SA phenotypes, analyses were performed with Trinculo (allelic model) and R *mlogit* package (recessive model), since multinomial regression is not implemented in PLINK. Accordingly, regarding gene-based tests, binary phenotypes recoded as “serious vs. none” and “recurrent vs. none”. Genetic correlations between SA phenotypes were estimated using Bayesian analysis^56^, using joint summary statistics to provide posterior probabilities that a given SNP has cross-associations with several phenotypes [*Cpbayes* R package^57^]; while also providing the Bayes Factor (BF) associated with the test to estimate the level of confidence in the associations^58^.

Adjusted regression models were then built for each SNP that was significantly associated with a given suicidal phenotype: binary logistic for the phenotype ‘any’ SA and multinomial regression for ‘serious’ and ‘recurrent’ SA. Other clinical and sociodemographic correlates of suicidal phenotypes were entered as cofactors or covariates. Collinearity between predictors was avoided by excluding variables with variance inflation factor (VIF) >2.5. Participants’ protocol of origin and gender were forcefully entered as potential confounders in each model. PLINK conditional regression was performed to identify the most relevant SNP in case several were associated with a given phenotype. Since we anticipated that most SNPs would show at least some interdependence (either due to their presence on a common and restricted pathway or due to high linkage disequilibrium [LD] effect), we chose family-wise error correction for multiple testing^59^. Permutation was preferred over Bonferroni correction, as previously suggested^60^. Adaptive permutation followed by the max (T) permutation correction set to 1,000 on the best association model (additive vs. genotypic vs. allelic vs. recessive) were used for SNP-based tests. Gene-based tests were corrected by adaptive permutation, followed by Bonferroni correction (see https://www.cog-genomics.org/plink/1.9/assoc#perm).

Hierarchical clustering, a method for unbiased classification of the data, was also used to assess how the different SA phenotypes would cluster together and with (i) clinically-relevant variables (gender, SUDs and CPD, number of psychotropic medications) and (ii) SNPs associated with any phenotype of SA according to the previous analysis steps. This method has been previously used for analyzing genetic associations in the case of multiple clinical endpoints that are likely to be interrelated^61^. The R package *pvclust* was used, allowing for both visualizing data clusters and estimating the significance of their constitutive variables after permutation^62^. Distance assessment used the D2-ward method. The *Unbiased Approximation* statistic was chosen to address the strength of support of a given cluster by the data, as recommended^62^, with a *p*-value ≤0.05 for considering significance.

Eventually, to further control for population stratification and estimate whether genetic differences would be associated with SA or with the sample status, we performed two exploratory regressions by comparing (i) patients vs. controls samples status patients as cases, whatever their SA history and (ii) comparing SA patients to controls. These analyses were performed with 55 shared SNPs of the ‘neurotrophic pathway’, using the same permutation parameters. All downstream statistical analyses were performed using R software version 3.5.3 through R studio 1.1.463^63^ running on Mac OS® X.12.6^64^. This study follows the *STREGA* guidelines for the report of genetic studies^65^. R session summary is available in the supplementary Fig. 2 (full R code available on demand from the authors).

### Methodology summary

- Merging two clinical samples of treatment-seeking outpatients suffering from cocaine or opioid use disorder on clinical, sociodemographic and genotype data;
- Genetic quality control (ancestry, relatedness, genotype missingness);
- Performing within-cases genetic association study on three suicidal phenotypes (‘any’, ‘recurrent’, ‘serious’) focusing on the ‘neurotrophic pathway’ (98 polymorphisms) at the SNP and the gene level, correcting for multiple testing and further adjusting for confounders;
- Strengthening/verifying genetic and clinical associations found in step 3 by hierarchical clustering;
- Discussing the functional significance of the genetic findings by using online databases;
- Looking for genetic correlation between ‘recurrent’ and ‘serious’ SA using Bayesian methods;
- Assessing the “signal-to-noise” ratio by investigating possible genetic differences between patients and controls regarding the ‘neurotrophic pathway’, centered on BDNF, regardless of the suicidal history.

## Results

### Clinical sample (Fig. 1 and Supplementary Table 2)

Patients were aged 39 +/−9 years and 78% were men (supplementary table 2 for sample description). There were 154 (37%) ‘any’ suicide attempters, 104 (68%) of whom reported at least one ‘serious’ SA and 96 (62%) of whom reported two SAs or more across the lifetime, thus being classified as ‘recurrent’ SAs. The median number of non-tobacco SUD was 3. Lifetime, ‘any’ suicide attempters were significantly more likely that non-suicide attempters to be women (OR = 2.05, *p* = 0.002), to be diagnosed with lifetime sedative use disorder (OR = 2.43, *p* < 0.001), currently smoke a higher number of cigarettes per day (OR = 1.05, *p* < 0.001), and have a number of lifetime SUD ≥ 3 (OR = 1.82, *p* = 0.002). ‘Serious’ and ‘recurrent’ suicide attempters shared the same clinical risk factors as ‘any’ lifetime suicide attempters (Supplementary Tables 3 & 4).

### SNP-based tests (Supplementary Fig. 1 and Supplementary Tables 5A–B)

Table 1 After permutations, two SNPs in the *BDNF* gene remained associated with ‘any’ lifetime SA (Supplementary Fig. 1) under the recessive model: rs7934165 (raw *p* = 2 × 10^−5^ and *p* [max(T)] = 0.0015, respectively) and rs10835210 (raw *p* = 0.0003, *p* [max(T)] = 0.0244). No SNP was associated with ‘serious’ or ‘recurrent’ SA under the genotypic or the recessive models. Minor allele homozygotes of the two SNPs associated with ‘any SA’ were distributed as follows: rs7934165 minor allele homozygotes, 33% (SA) vs. 14% (no SA), *p* < 0.001, and rs10835210, 26% (SA) vs. 11% (no SA), *p* < 0.001. No other gene harbored any SNP associated with SA, whatever the model and the phenotype considered. Summary statistics for the three suicidal phenotypes under the recessive model are available in the supplementary Tables 5A–B.

**Table 1:** Top SNP association of each suicidal phenotype under the recessive model.

CHR	POS (bp)	Gene and SNP ID	Corrected *p*-value	Variant properties	CADD	Significant QTL	RegulomeDB	MAF (EUR)	MAF (sample)	Effect direction
Any SA, lifetime										
11	27731983	*BDNF* rs7934165	0.0244*	Intron variant	14.38	E0, M7	NA	0.47	0.48	↑
11	27695910	*BDNF* rs10835210	0.0015*	Intron variant	17.18	E1, M0	3a	0.44	0.42	↑
At least one serious SA, lifetime										
9	87590382	*NTRK2* rs4877894	0.868	Intron variant	NA	E0, M0	NA	0.2	0.44	↑
≥2 SAs, lifetime										
15	88671372	*NTRK3* rs1104765	0.6871	Intron variant	NA	E0, M0	4	0.28	0.25	↑

*CHR* chromosome, *POS* genomic coordinates in base pairs (bp), *CADD* combined annotation dependent depletion, *QTLs* quantitative trait loci (E for expression data with the corresponding number of significant loci, *M* for methylation data with the corresponding number of significant CpG sites), *MAF* minor allele frequency. *p < 0.05 after 1000 permutations.

Bold values indicate *p* values < 0.05.

### Gene-based tests

Gene-based testing confirmed the results of SNP-based tests, the *BDNF* gene being associated with ‘any’ lifetime SA due to both rs7934165 and the ValMet66 SNP rs6525 (*p* = 0.0014 after 12,753 permutations, Bonferroni-corrected *p* = 0.014 for 4 sets selected for harboring at least one significant SNP, out of 11 genes sets tested, after adjustment for gender, CPD and protocol of origin).

### Adjusted regression models

**Fig. 2:**
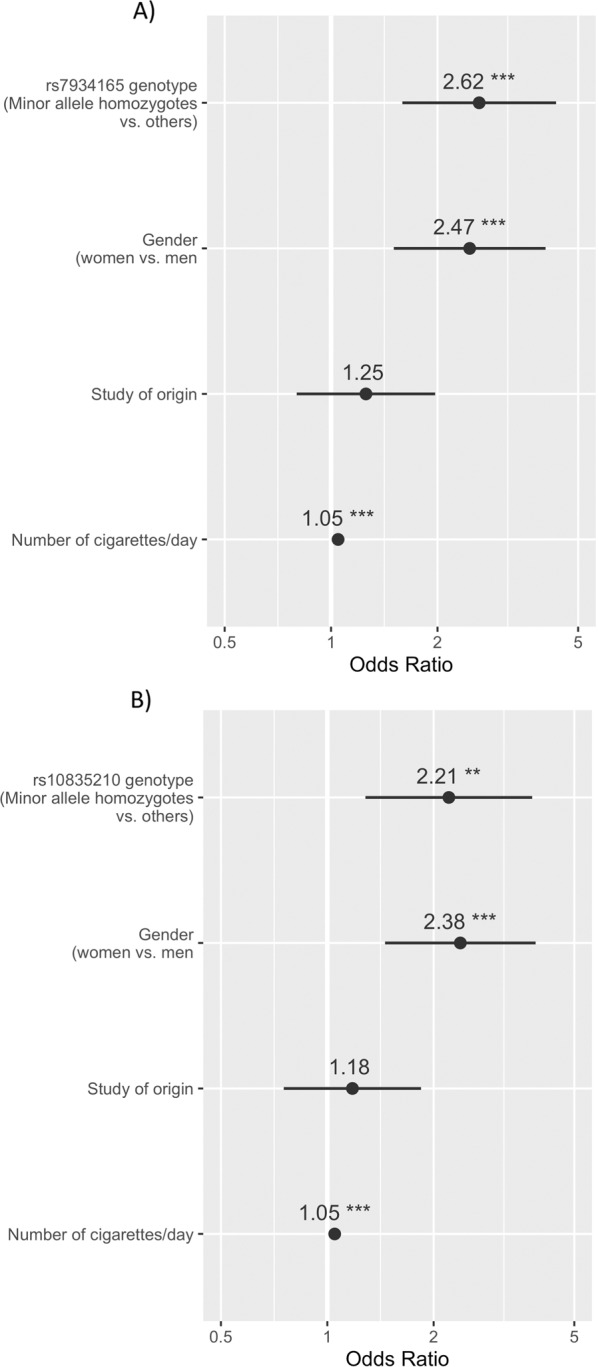
Plots for logistic regression with ‘any’ SA as a dependent variable, sociodemographic and clinical confounders and (A) rs7934165 or (B) rs10835210BDNF polymorphisms as independent variables. Odds ratios are displayed. Bars indicate 95% confidence intervals. *p < 0.05, **p < 0.01, and ***p < 0.001.

(Fig. 2) After adjustment for covariates, under the recessive model, rs7934165 and rs10835210 remained associated with lifetime SA (OR = 2.62 and OR = 2.21, respectively; both *p* < 0.001). The number of CPD (both ORs = 1.05) and female gender (ORs = 2.47 when testing rs7934165 and 2.38 when testing rs10835210) remained significantly associated with lifetime SA in both the regression models. When conditioning the regression on the other significant SNP, the associations failed to reach significance, while noticing that these are in full LD (*r*^2^ = 1).

### Classification by hierarchical clustering

**Fig. 3:**
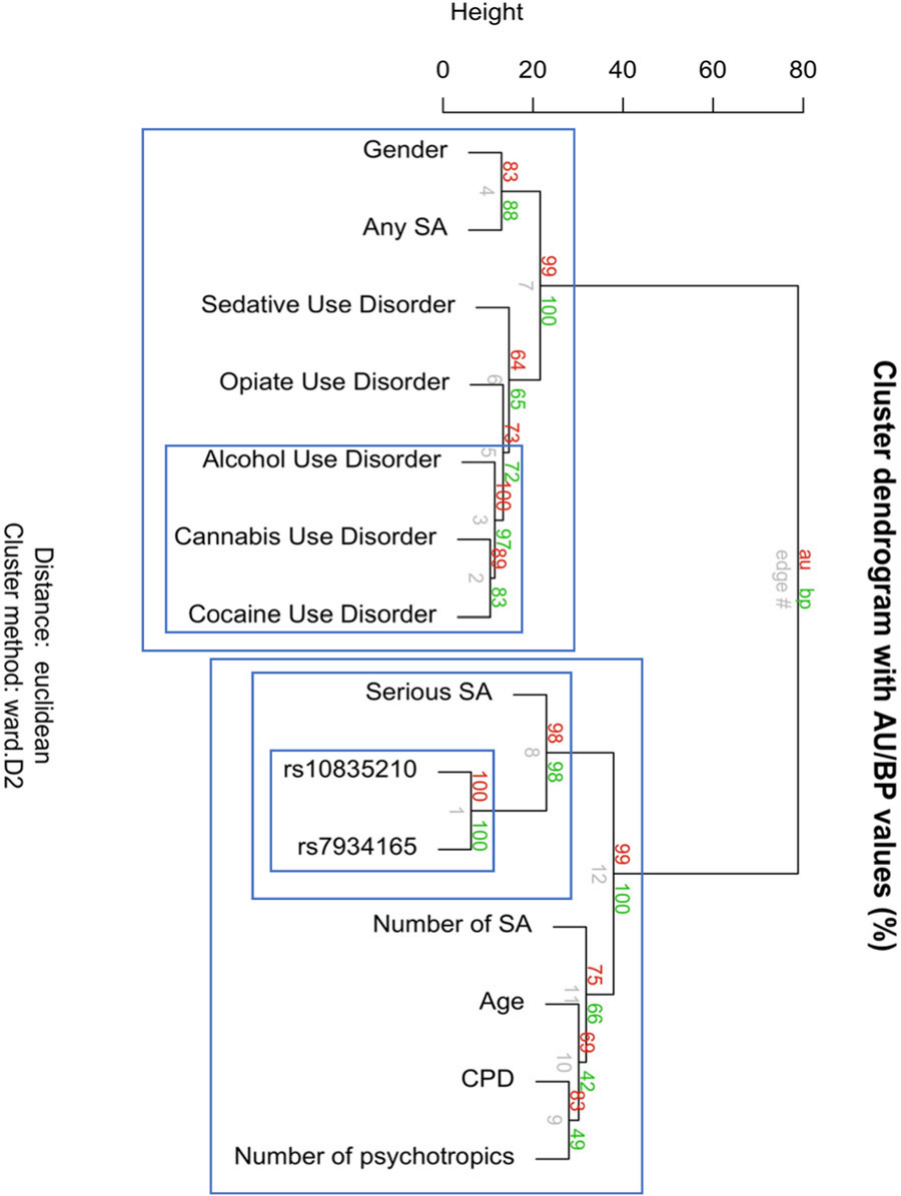
Hierarchical clustering. Cluster dendogram using euclidian distance and D2, ward clustering of all variables associated with SA to some extent according to bivariate analyses.

The dendrogram elicited two main clusters, which were significant (Fig. 3). The first one encompassed ‘any’ SA, gender, and SUD diagnoses, composed by two significant branches and including a significant cluster of alcohol use and cocaine + cannabis use disorders. The second one was also composed by two significant branches, including ‘serious’ SA and *BDNF* SNPs rs7934165 and rs10835210 on the one hand and the number of lifetime, ‘any’ SA, age, the number of cigarettes per day, the number of psychotropic medication on the other hand. Strikingly in this cluster, ‘serious’ SA and *BDNF* SNPs rs10835210 and rs7934165 (the two SNPs associated with ‘any' SA) further represented a significant cluster.

### Functional implication of the genetic associations (Table 1)

*BDNF* rs7934165 has a CADD score of 14.38, is not scored in the *regulomeDB* and has no significant influence on *BDNF* expression in the brain according to the *GTEx portal* database (lowest *p*-value = 0.031 for the putamen area, not significant after correction for multiple testing). rs10835210 has a CADD score of 17.18, is scored 3a in *regulomeDB* (less likely to affect binding), and is significantly associated with increased levels of BDNF antisense (AS) mRNA by increasing expression of the *BDNF-AS* gene in the frontal cortex (normalized effect size = 0.24).

### Pleiotropy

According to Bayesian genetic correlation analysis, rs7934165 had posterior probabilities of association with ‘any’ SA, ‘serious’ SA and ‘recurrent’ SA of 98%, 81%, and 57%, respectively. BF was 4.8. As for rs10835210, posterior probabilities were 90%, 32%, and 23%, respectively, with BF = 15.2.

### Comparisons with the control sample

None of the 55/98 variants shared by the two samples in the ‘neurotrophic pathway’ were significantly different in the clinical vs. control samples. rs7934165 had a minor allele frequency of 48% in SUD patients and 47% in controls (Chi^2^ = 0.007, *p* = 0.933). rs10835210 was not genotyped in controls. There were 405 SNPs with significantly different frequencies between samples (*p*-values <0.05 after 1000 permutations, supplementary table 6).

## Discussion

This study on the genetics of the ‘neurotrophic pathway’ and SA in patients with multiple SUDs showed the *BDNF* gene to have consistent SNP-based and gene-based associations with lifetime, ‘any’ SA. Associations between SA and *BDNF* SNPs rs7934165 and rs10835210 involved changes in *BDNF* expression and methylation, withstood multiple adjustments, and were supported by Bayesian analysis. The latter further showed pleiotropy of rs7934165 with both ‘any’ and ‘serious’ SA. Unbiased data classification by hierarchical clustering indicated that both *BDNF* SNPs clustered together with ‘serious’ SA.

Our group previously reported associations between SA and female gender, tobacco smoking and sedative use disorders in patients with multiple SUDs^4^. Furthermore, the association between SA and female gender was also shown in other, independent samples of patients seeking treatment for SUD^66,67^. Female gender thus seems to remain an independent and strong risk factor for SA, regardless of the population studied. However, in this study, other consistent associations previously reported between ‘serious’ SA and male gender and between ‘recurrent’ SA and female gender (see e.g., ref. ^68^ for a nationwide registry study on the subject and^69^ for a review paper), were not replicated. This suggests that clinical and/or genetic factors may have overcome these gender effects, acknowledging that our female subsample was relatively small. ‘Recurrent’ and ‘serious’ SA clustered separately from ‘any’ SA, in line with previous findings^5^. Bayesian analysis showed that rs7934165 has a modest but strongly shared association with both ‘any’ and ‘serious’ SA, while the association between rs10835210 and ‘any’ SA was strong but not shared with the other suicidal phenotypes. Here, there is evidence that patients with multiple SUDs may also carry innate liability to SA related to BDNF, which appears to be shared between ‘any’ and ‘serious’ SA.

Given the function of the BDNF, this liability is likely to interact with multiple distal and proximal stressors to increase SA risk^8^. From a strict genetic perspective, the associations shown in this study were strongest under a recessive model, which raises several issues. First, given that the sample size was modest, the lack of significant association under the genotypic model could be a false negative. BF for the genotypic model was inconclusive at 2.45 when analyzing the three suicidal phenotypes. Second, the genotypic model is better suited for SNPs with large effect sizes. The recessive model fits with the multigenic theory of complex traits such as psychiatric disorders, which states that SNPs are likely to have weak effects on the trait (or on the pathway to a trait)^70^, as opposed to severe neurodevelopmental or metabolic disease. It is noteworthy that, in a meta-analysis of association studies between *BDNF* 166ValMet functional polymorphism and suicidal behavior, only the recessive model yielded a significant result [Fig. 1C from ref. ^71^].

The two SNPs associated with SA in this study are in very strong LD (*r*^2^ = 1). Both further clustered with ‘serious’ SA (Fig. 3). From a functional perspective, both associations are potential modifiers of BDNF activity. First, the *BDNF* gene is highly susceptible to methylation, which has been associated with suicidal behavior [at least in the promoter/exon IV region^72,73^], and the SNP rs7934165 is associated with increased *cis* methylation at seven Cpg sites suggesting a decreased expression level. There was consistent evidence that this SNP shared its associations between ‘any’ and ‘serious’ SA, given results from Bayesian analysis and hierarchical clustering, despite the absence of association with ‘serious’ SA itself analyzed separately. The association of rs10835210 is of particular interest because its risk allele is associated with increased expression of the *BDNF-AS* gene. This gene encodes an antisense RNA that represses BDNF transcription by altering chromatin structure at the *BDNF* locus, thus reducing endogenous BDNF protein and function^74^. Psychiatric studies have often focused on plasma BDNF levels, which do not seem to be associated with SA^24^^.^ However, there is emerging evidence that major cellular mediators of the neuronal impact of BDNF show functional impairments in major depression^75^ and suicidal behavior ^76^. Taken altogether, both associations are likely to exert a synergistic effect toward a time- and possibly event-specific reduction in *BDNF* level, bearing in mind that neither SNP alone was associated with SA when conditioning the adjusted regression on the other.

Limitations must be acknowledged. Data collection was retrospective, which may introduce recall bias including for SA assessment. Data from two similar, yet not identical protocols was pooled and the primary inclusion criterion for this study was a diagnosis of cocaine or opiate use disorder, which may have introduced some heterogeneity at both the clinical and the genetic levels. However, we did not find any association between the protocol of origin and *BDNF* SNPs, whilst available evidence suggest that both opioid and cocaine use disorders have been associated with decreased plasma BDNF levels^77–79^, but not with *BDNF* polymorphisms^77,80^. Thus it seems unlikely that the primary SUD diagnosis could have biased our genetic association results. There were variable missing data rates in the clinical variables collected in this study. We did not record the proportions of proposed/eligible/included participants to address representativeness. Non-addictive psychiatric disorders were not ascertained through standardized questionnaires, so that the relationship between *BDNF* and SA could have been confounded by conditions such as major depression^81^. However, although using patients’ ongoing treatment as proxies for depressive or anxiety disorders is subject to caution, one must note that SA was not associated with major depression in a previous study of methadone-maintained patients assessed with a structured interview^82^. The definition of ‘recurrent’ SA was arbitrary, and further validation of a cutoff in the current sample could help identifying more specific risk factors. Finally, the lack of data regarding childhood trauma and impulsivity is a potentially important limitation of this study. Sexual abuse notably increases the risk for SA in populations with mental disorders^83^, especially for ‘recurrent’ SA^84^, possibly in interaction with variations in the *BDNF* gene^22,85^. However, the latter associations rather regarded depression and anxiety than SA itself^24,72^. Impulsiveness has also been associated with SA^86^. It is particularly elevated in high-risk psychiatric samples^87,88^, amongst whom it has shown inconsistent associations with SA^89^, suggesting the presence of some ceiling effect.

The study has several strengths. First of all, thorough quality check was performed as regards genetic data, which included genetic-based identification of related individuals and of Caucasian ancestry. Accordingly, we used stringent procedures to control for multiple testing, regarding both the genetic and the phenotypic data. A relevant pathway repeatedly associated with SA in the literature was studied and the phenotypic characterization was extensive. Complementary analytic methods were used to test our initial hypothesis, yielding mostly convergent results. There was no genetic difference between outpatients with opiate or cocaine use disorder and UK blood donors, further supporting the absence of bias related to population stratification in our results regarding *BDNF* and the ‘neurotrophic pathway’.

Overall, this study shed light on the pathophysiology of suicidal behavior in patients with multiple SUDs in a clinically-representative sample. Importantly, although the sample was recruited in tertiary care centers, such clinical settings are organized to remain as accessible as primary care centers to treatment-seeking individuals, even if they are hospital-based, and they must warrant both anonymity and free access^90^. A major result was that ‘serious’ SA shared both clinical and genetic risk factors with SA—not otherwise specified. Given that 68% suicide attempters reported ‘serious’ SA in our sample, this suggests that these suicidal phenotypes share some level of genetic liability in this multiple SUDs population, strongly involving the stress-related neurobiological pathway modulating effects of BDNF. Although the BDNF and the ‘neurotrophic pathway’ have been associated with a wide range of psychopathology, we tentatively propose that it could open avenues toward the identification of specific biomarkers of more severe suicidal profiles in high-risk populations, such as those with severe/multiple SUDs. This interpretation comes from the fact that most individuals in the current sample carry, overall, a high-risk load for SA, and that no genetic association with current psychotropic medication—a proxy for psychopathology—was found. Therefore, detecting biological differences between subgroups of individuals can be regarded as relatively specific for SA, and may have been facilitated by the use of SA subphenotypes^91^. Such biomarkers for SA risk should be properly tested for their predictive power in prospective studies^92^ and combined with state-dependent markers at the biological and clinical levels, as was successfully reported in major depression^92^. The clinical utility of such markers would be high: increasing the monitoring of suicidal ideation and associated symptoms such as hopelessness, and focusing on substances of abuse that have been associated with SA in the SUD population (even if they were not the primary focus of the patient or the clinician). This would also contribute to the global effort to reduce suicide mortality, which remains a priority in most countries worldwide^45,93^, especially given that studies distinguishing severity profiles of SA in SUD populations remain scarce.

## Supplementary information

Supplementary Table 1

Supplementary Table 5A

Supplementary Table 5B

Supplementary Table 6

SUpplementary Figure 2

Supplementary Figure 1

Supplementary Tables 2 to 4
