# Supplementary material for: Clustering suicidal phenotypes and genetic associations with brain-derived neurotrophic factor in patients with substance use disorders
Source: Transl Psychiatry. 2021 Jan 21;11:72. doi: 10.1038/s41398-021-01200-5 (PMC7820499; doi:10.1038/s41398-021-01200-5)
Supplement: Supplementary file 1 — Supplementary Table 1 [file 41398_2021_1200_MOESM1_ESM.pdf]

Supplementary Table 1: names of all markers from the BDNF pathway present on the DNA array and their correspondence with dbSNP 150.
Markers, which were selected for the study are indicated by *.

Name	RsID	Gene	gene_length	coverage_ratio	number_SNP	start
exm897559		BDNF	67165	0.25	17	27679649
exm897569	rs139352447	BDNF	67165	0.25	17	27679889
exm897570	rs6265*	BDNF	67165	0.25	17	27679916
exm897574	rs8192466	BDNF	67165	0.25	17	27680107
exm897586	rs66866077*	BDNF	67165	0.25	17	27720937
psy_rs117853517	rs117853517	BDNF	67165	0.25	17	27736029
psy_rs34043390	rs34043390	BDNF	67165	0.25	17	27702915
rs10835210	rs10835210*	BDNF	67165	0.25	17	27695910
rs10835211	rs10835211*	BDNF	67165	0.25	17	27701365
rs12291063	rs12291063	BDNF	67165	0.25	17	27694101
rs2030323	rs2030323*	BDNF	67165	0.25	17	27728539
rs6265	rs6265*	BDNF	67165	0.25	17	27679916
rs7934165	rs7934165*	BDNF	67165	0.25	17	27731983
var_11_27695731		BDNF	67165	0.25	17	27695732
indel-17303		BDNF	67165	0.25	17	27695787
variant-17304		BDNF	67165	0.25	17	27720928
variant-17305		BDNF	67165	0.25	17	27721029
exm2218869	rs6266	CNTF	38691	0.18	7	58391937
exm912709	rs75769623	CNTF	38691	0.18	7	58390293
exm912711	rs139149169	CNTF	38691	0.18	7	58390308
exm912717	rs17152779	CNTF	38691	0.18	7	58391538
exm912723	rs147908441	CNTF	38691	0.18	7	58391584
exm912728	rs142784651	CNTF	38691	0.18	7	58391682
exm912730	rs143626643	CNTF	38691	0.18	7	58391696
exm912743	rs145968911	CNTF	38691	0.18	7	58391957
newrs1800169	rs1800169*	CNTF	38691	0.18	7	58391501
indel-18300		CNTF	38691	0.18	7	58391789
exm747193		CNTFR	38691	0.18	7	34557610
exm747203	rs145830233	CNTFR	38691	0.18	7	34564628
exm747205	rs200077465	CNTFR	38691	0.18	7	34564654
exm747208	rs115175006	CNTFR	38691	0.18	7	34564707

rs10758268	rs10758268*	CNTFR	38691	0.18	7	34572767
rs4879805	rs4879805*	CNTFR	38691	0.18	7	34564274
rs7044318	rs7044318*	CNTFR	38691	0.18	7	34554999
exm1334492	rs140781803	NGFR	1737	0.58	1	47583847
exm1334503	rs149305371	NGFR	1737	0.58	1	47587809
exm1334504	rs2072446*	NGFR	1737	0.58	1	47587819
exm1334510	rs148524226	NGFR	1737	0.58	1	47589294
exm1334514		NGFR	1737	0.58	1	47589334
exm1334523	rs143067054	NGFR	1737	0.58	1	47590238
exm1891268	rs114337127	NGFR	1737	0.58	1	47579431
exm1891270	rs115146121	NGFR	1737	0.58	1	47583681
exm1891277	rs191965251	NGFR	1737	0.58	1	47587995
psy_rs11466155	rs11466155	NGFR	1737	0.58	1	47588000
psy_rs141373032	rs141373032	NGFR	1737	0.58	1	47579806
rs3785931	rs3785931*	NGFR	1737	0.58	1	47578118
exm2202370	rs182219749	NGFRAP1	1737	0.58	1	102632714
exm2251076	rs6332*	NTF3	89424	0.16	14	5603632
exm976252	rs151156814	NTF3	89424	0.16	14	5603540
exm976274	rs199928382	NTF3	89424	0.16	14	5603970
exm976276	rs200719143	NTF3	89424	0.16	14	5603981
rs10774329	rs10774329*	NTF3	89424	0.16	14	5554678
rs10774330	rs10774330*	NTF3	89424	0.16	14	5557893
rs10774336	rs10774336*	NTF3	89424	0.16	14	5626158
rs10774339	rs10774339*	NTF3	89424	0.16	14	5630281
rs11063699	rs11063699*	NTF3	89424	0.16	14	5587152
rs11063708	rs11063708*	NTF3	89424	0.16	14	5594403
rs11063723	rs11063723*	NTF3	89424	0.16	14	5618814
rs11612899	rs11612899*	NTF3	89424	0.16	14	5613536
rs7958038	rs7958038*	NTF3	89424	0.16	14	5576594
rs7974186	rs7974186*	NTF3	89424	0.16	14	5575467
exm1489423	rs121918427	NTF4	9283	0.32	3	49564639
exm1489442	rs61732310	NTF4	9283	0.32	3	49564992

exm2229747		NTF4	9283	0.32	3	49564629
exm110370	rs146718843	NTRK1	66210	0.98	65	156785800
exm110378	rs142614264	NTRK1	66210	0.98	65	156786487
exm110391	rs35627952	NTRK1	66210	0.98	65	156810763
exm110393	rs141650660	NTRK1	66210	0.98	65	156810789
exm110400	rs144096948	NTRK1	66210	0.98	65	156811202
exm110403	rs149108213	NTRK1	66210	0.98	65	156811231
exm110405	rs142758140	NTRK1	66210	0.98	65	156811255
exm110416	rs201172210	NTRK1	66210	0.98	65	156811515
exm110426	rs147703846	NTRK1	66210	0.98	65	156811909
exm110430	rs200176126	NTRK1	66210	0.98	65	156811994
exm110435	rs201715497	NTRK1	66210	0.98	65	156812213
exm110437	rs140531495	NTRK1	66210	0.98	65	156812236
exm110440	rs138327752	NTRK1	66210	0.98	65	156812296
exm110465		NTRK1	66210	0.98	65	156814385
exm110469	rs199807142	NTRK1	66210	0.98	65	156814548
exm110471	rs200802232	NTRK1	66210	0.98	65	156814608
exm110473	rs150115105	NTRK1	66210	0.98	65	156814868
exm110489	rs149045422	NTRK1	66210	0.98	65	156815411
exm110501	rs200595391	NTRK1	66210	0.98	65	156815806
exm110515	rs138923152	NTRK1	66210	0.98	65	156816335
exm110529	rs201179362	NTRK1	66210	0.98	65	156819094
exm110534	rs148814291	NTRK1	66210	0.98	65	156819165
exm110562	rs143605369	NTRK1	66210	0.98	65	156821759
exm110572	rs56377825	NTRK1	66210	0.98	65	156821885
exm110573	rs55951840	NTRK1	66210	0.98	65	156821890
exm110595	rs55757706	NTRK1	66210	0.98	65	156823801
exm110596	rs140557452	NTRK1	66210	0.98	65	156823805
exm110617	rs143369504	NTRK1	66210	0.98	65	156823976
exm110620	rs140386495	NTRK1	66210	0.98	65	156824054
exm110623	rs140915043	NTRK1	66210	0.98	65	156828358
exm110633	rs139140006	NTRK1	66210	0.98	65	156834169

exm110635	rs79678945	NTRK1	66210	0.98	65	156834187
exm110644	rs201509045	NTRK1	66210	0.98	65	156834527
exm110655	rs41267427	NTRK1	66210	0.98	65	156837904
exm110660	rs150271893	NTRK1	66210	0.98	65	156837949
exm110668	rs146201511	NTRK1	66210	0.98	65	156838324
exm110669	rs148324672	NTRK1	66210	0.98	65	156838333
exm110672	rs201192875	NTRK1	66210	0.98	65	156838353
exm110688	rs137979116	NTRK1	66210	0.98	65	156843439
exm110692	rs137994522	NTRK1	66210	0.98	65	156843514
exm110698	rs139875058	NTRK1	66210	0.98	65	156843595
exm110721	rs56320207	NTRK1	66210	0.98	65	156844777
exm110723	rs34900547	NTRK1	66210	0.98	65	156844800
exm110726	rs144901788	NTRK1	66210	0.98	65	156845431
exm110740	rs150579345	NTRK1	66210	0.98	65	156846300
exm110750	rs6336	NTRK1	66210	0.98	65	156848918
exm110751	rs6339	NTRK1	66210	0.98	65	156848946
exm110768	rs199905593	NTRK1	66210	0.98	65	156849792
exm110781	rs145081333	NTRK1	66210	0.98	65	156851265
exm110788	rs35669708	NTRK1	66210	0.98	65	156851382
exm1719390	rs141592864	NTRK1	66210	0.98	65	156834558
exm1719411	rs199826686	NTRK1	66210	0.98	65	156844404
exm1719422	rs200575096	NTRK1	66210	0.98	65	156845892
exm2231495	rs2274496	NTRK1	66210	0.98	65	156834198
exm2250011	rs201311148	NTRK1	66210	0.98	65	156824007
exm2273701	rs55892037	NTRK1	66210	0.98	65	156846256
psy_rs11264574	rs11264574	NTRK1	66210	0.98	65	156826574
psy_rs4661064	rs4661064	NTRK1	66210	0.98	65	156825004
psy_rs72698666	rs72698666	NTRK1	66210	0.98	65	156834364
rs1800601	rs1800601*	NTRK1	66210	0.98	65	156785617
rs4661061	rs4661061*	NTRK1	66210	0.98	65	156802657
rs7522395	rs7522395*	NTRK1	66210	0.98	65	156796697
rs7534418	rs7534418*	NTRK1	66210	0.98	65	156805803

indel-7462		NTRK1	66210	0.98	65	156812800
variant-7482		NTRK1	66210	0.98	65	156844801
exm2266843	rs1187350*	NTRK2	355039	0.12	43	87295237
exm758498	rs78936193	NTRK2	355039	0.12	43	87285694
exm758511	rs150692457	NTRK2	355039	0.12	43	87322819
exm758515	rs76950094	NTRK2	355039	0.12	43	87325623
exm758522	rs117250170	NTRK2	355039	0.12	43	87338511
exm758523	rs139913267	NTRK2	355039	0.12	43	87338515
exm758549	rs78629699	NTRK2	355039	0.12	43	87359924
exm758555	rs145968424	NTRK2	355039	0.12	43	87359980
exm758585	rs201902834	NTRK2	355039	0.12	43	87636209
psy_rs112018820	rs112018820	NTRK2	355039	0.12	43	87463565
psy_rs140010892	rs140010892	NTRK2	355039	0.12	43	87474115
rs10512154	rs10512154*	NTRK2	355039	0.12	43	87441475
rs10780691	rs10780691*	NTRK2	355039	0.12	43	87491253
rs10868238	rs10868238*	NTRK2	355039	0.12	43	87530935
rs10868241	rs10868241*	NTRK2	355039	0.12	43	87593028
rs11140745	rs11140745*	NTRK2	355039	0.12	43	87355358
rs1187286	rs1187286*	NTRK2	355039	0.12	43	87415028
rs1187287	rs1187287*	NTRK2	355039	0.12	43	87414794
rs1187337	rs1187337*	NTRK2	355039	0.12	43	87316037
rs1187343	rs1187343*	NTRK2	355039	0.12	43	87308783
rs1187350	rs1187350*	NTRK2	355039	0.12	43	87295237
rs12340212	rs12340212*	NTRK2	355039	0.12	43	87551964
rs12685376	rs12685376	NTRK2	355039	0.12	43	87505172
rs1387924	rs1387924*	NTRK2	355039	0.12	43	87632993
rs1573219	rs1573219*	NTRK2	355039	0.12	43	87387622
rs1619120	rs1619120*	NTRK2	355039	0.12	43	87302196
rs17080621	rs17080621	NTRK2	355039	0.12	43	87398104
rs1838158	rs1838158*	NTRK2	355039	0.12	43	87473009
rs1899640	rs1899640*	NTRK2	355039	0.12	43	87409025
rs1948308	rs1948308*	NTRK2	355039	0.12	43	87616257

rs2083828	rs2083828*	NTRK2	355039	0.12	43	87447045
rs2808707	rs2808707*	NTRK2	355039	0.12	43	87558294
rs3739804	rs3739804*	NTRK2	355039	0.12	43	87421631
rs3860945	rs3860945*	NTRK2	355039	0.12	43	87585624
rs4361832	rs4361832*	NTRK2	355039	0.12	43	87595734
rs4877894	rs4877894*	NTRK2	355039	0.12	43	87590382
rs6559838	rs6559838*	NTRK2	355039	0.12	43	87553563
rs6559840	rs6559840*	NTRK2	355039	0.12	43	87575500
rs7048015	rs7048015*	NTRK2	355039	0.12	43	87478135
rs716893	rs716893*	NTRK2	355039	0.12	43	87419117
rs7855888	rs7855888*	NTRK2	355039	0.12	43	87452058
rs7855989	rs7855989	NTRK2	355039	0.12	43	87556420
rs923559	rs923559*	NTRK2	355039	0.12	43	87616532
chr15_88423539		NTRK3	381769	0.15	59	88423539
exm1186142	rs139392904	NTRK3	381769	0.15	59	88472622
exm1186164	rs141300787	NTRK3	381769	0.15	59	88522610
exm1186208	rs145157285	NTRK3	381769	0.15	59	88678541
exm1186213	rs148888023	NTRK3	381769	0.15	59	88678619
exm1186228	rs142726068	NTRK3	381769	0.15	59	88680655
exm1186240	rs149623569	NTRK3	381769	0.15	59	88690618
exm1186256	rs200822610	NTRK3	381769	0.15	59	88799324
exm2252355	rs201918746	NTRK3	381769	0.15	59	88576170
exm2252356	rs202151145	NTRK3	381769	0.15	59	88680654
exm2252357	rs3784404*	NTRK3	381769	0.15	59	88688097
exm2264582	rs8025146*	NTRK3	381769	0.15	59	88737834
psy_rs117333861	rs117333861	NTRK3	381769	0.15	59	88608988
psy_rs1435397	rs1435397*	NTRK3	381769	0.15	59	88454826
psy_rs146797905	rs146797905	NTRK3	381769	0.15	59	88758574
psy_rs17828501	rs17828501	NTRK3	381769	0.15	59	88623784
psy_rs72756147	rs72756147	NTRK3	381769	0.15	59	88509896
psy_rs8024898	rs8024898*	NTRK3	381769	0.15	59	88482578
rs10163131	rs10163131	NTRK3	381769	0.15	59	88439774

rs1104765	rs1104765*	NTRK3	381769	0.15	59	88671372
rs1107292	rs1107292*	NTRK3	381769	0.15	59	88758621
rs11073755	rs11073755*	NTRK3	381769	0.15	59	88523321
rs11073767	rs11073767*	NTRK3	381769	0.15	59	88706936
rs11631508	rs11631508*	NTRK3	381769	0.15	59	88498466
rs11635443	rs11635443*	NTRK3	381769	0.15	59	88771571
rs11636250	rs11636250*	NTRK3	381769	0.15	59	88658964
rs11855377	rs11855377*	NTRK3	381769	0.15	59	88463831
rs11857753	rs11857753	NTRK3	381769	0.15	59	88747766
rs12148845	rs12148845*	NTRK3	381769	0.15	59	88703546
rs12594283	rs12594283*	NTRK3	381769	0.15	59	88584252
rs13380271	rs13380271*	NTRK3	381769	0.15	59	88499185
rs1369430	rs1369430*	NTRK3	381769	0.15	59	88430769
rs1560975	rs1560975*	NTRK3	381769	0.15	59	88423463
rs16941103	rs16941103*	NTRK3	381769	0.15	59	88525951
rs16941241	rs16941241	NTRK3	381769	0.15	59	88630705
rs16941334	rs16941334*	NTRK3	381769	0.15	59	88699342
rs1946697	rs1946697*	NTRK3	381769	0.15	59	88465057
rs2018052	rs2018052*	NTRK3	381769	0.15	59	88519216
rs2117655	rs2117655*	NTRK3	381769	0.15	59	88428702
rs2349057	rs2349057*	NTRK3	381769	0.15	59	88665139
rs3784413	rs3784413	NTRK3	381769	0.15	59	88653595
rs3825885	rs3825885*	NTRK3	381769	0.15	59	88602841
rs4887350	rs4887350*	NTRK3	381769	0.15	59	88593449
rs4887368	rs4887368*	NTRK3	381769	0.15	59	88676679
rs4887381	rs4887381*	NTRK3	381769	0.15	59	88737328
rs4887399	rs4887399*	NTRK3	381769	0.15	59	88790325
rs4887400	rs4887400*	NTRK3	381769	0.15	59	88794859
rs6496466	rs6496466*	NTRK3	381769	0.15	59	88717708
rs6496469	rs6496469*	NTRK3	381769	0.15	59	88735310
rs7176429	rs7176429*	NTRK3	381769	0.15	59	88419424
rs8030107	rs8030107*	NTRK3	381769	0.15	59	88547290

rs8031871	rs8031871*	NTRK3	381769	0.15	59	88493008
rs8033396	rs8033396	NTRK3	381769	0.15	59	88742439
rs8035239	rs8035239*	NTRK3	381769	0.15	59	88674990
rs8041239	rs8041239*	NTRK3	381769	0.15	59	88475356
rs9806762	rs9806762*	NTRK3	381769	0.15	59	88661739
variant-37307		NTRK3	381769	0.15	59	88474442
variant-37308		NTRK3	381769	0.15	59	88476238
variant-37310		NTRK3	381769	0.15	59	88483946
exm1341222	rs202177251	SKA2	45318	0.18	8	57189698
exm1341229	rs112928700	SKA2	45318	0.18	8	57196822
exm1341240	rs77282282	SKA2	45318	0.18	8	57232365
rs11654986	rs11654986	SKA2	45318	0.18	8	57206124
rs8077052	rs8077052	SKA2	45318	0.18	8	57208921
rs9911583	rs9911583	SKA2	45318	0.18	8	57210721
indel-47643		SKA2	45318	0.18	8	57196720
variant-47644		SKA2	45318	0.18	8	57208637
exm81101	rs72646588	SORT1	88381	0.2	18	109859485
exm81105	rs150416069	SORT1	88381	0.2	18	109859580
exm81112	rs72646577	SORT1	88381	0.2	18	109865630
exm81122	rs149226217	SORT1	88381	0.2	18	109867673
exm81135	rs144141753	SORT1	88381	0.2	18	109878893
exm81136	rs2228606	SORT1	88381	0.2	18	109878903
exm81138	rs144973301	SORT1	88381	0.2	18	109878920
exm81143	rs200994282	SORT1	88381	0.2	18	109883391
exm81148	rs202245759	SORT1	88381	0.2	18	109883405
exm81154	rs2228605	SORT1	88381	0.2	18	109884672
exm81161	rs141749679	SORT1	88381	0.2	18	109888432
exm81167	rs149456022	SORT1	88381	0.2	18	109897022
exm81172	rs140106626	SORT1	88381	0.2	18	109897119
exm81178	rs61797119	SORT1	88381	0.2	18	109910100
rs11102972	rs11102972*	SORT1	88381	0.2	18	109880721
rs12037569	rs12037569*	SORT1	88381	0.2	18	109923677

rs464218	rs464218*	SORT1	88381	0.2	18	109856306
indel-5426		SORT1	88381	0.2	18	109865570
