# Supplementary material for: Clustering suicidal phenotypes and genetic associations with brain-derived neurotrophic factor in patients with substance use disorders
Source: Transl Psychiatry. 2021 Jan 21;11:72. doi: 10.1038/s41398-021-01200-5 (PMC7820499; doi:10.1038/s41398-021-01200-5)
Supplement: Supplementary file 2 — Supplementary Table 5A [file 41398_2021_1200_MOESM2_ESM.pdf]

Supplementary Table 5A: summary statistics of genetic associations between the BDNF pathway and suicide attempts (“not specified” and “serious”).

							Any lifetime SA				***At least one serious SA (multinomial regression with no SA as the reference variable)***						
												One SA but not serious			*At least one serious SA*		
CHROM	POS	rsID	gene	A1	A2	N	TEST	SE	Z_STAT	P	TEST	SE	Z	P	SE	Z	P
1	109856306	rs464218	*SORT1*	G	A	411	REC	0.265351	0.541764	0.587981	ALL	0.2281	-1.22642	0.220042	0.170563	-1.18985	0.234105
1	109880721	rs11102972	*SORT1*	G	A	411	REC	0.453236	0.52256	0.601281	ALL	0.266406	-0.00609624	0.995136	0.193998	-0.627448	0.530366
1	109923677	rs12037569	*SORT1*	A	C	411	REC	1.08498	-1.19518	0.232019	ALL	0.308863	-0.453444	0.650229	0.229875	-0.775757	0.437893
1	156785617	rs1800601	*NTRK1*	G	A	411	REC	0.329566	-0.281021	0.778694	ALL	0.243283	1.27021	0.20401	0.177312	1.14417	0.252554
1	156796697	rs7522395	*NTRK1*	A	G	411	REC	0.407332	0.940471	0.346976	ALL	0.264186	0.809568	0.418188	0.193434	0.883252	0.3771
1	156802657	rs4661061	*NTRK1*	A	G	411	REC	0.361516	0.833733	0.404432	ALL	0.24945	0.921696	0.356687	0.186821	1.49379	0.13523
1	156805803	rs7534418	*NTRK1*	A	G	411	REC	0.31232	0.638481	0.523161	ALL	0.235999	0.875243	0.381442	0.173033	0.756814	0.449161
9	34554999	rs7044318	*CNTFR*	A	G	411	REC	0.565933	0.65396	0.513137	ALL	0.30207	0.828759	0.407241	0.206409	-0.703298	0.48187
9	34564274	rs4879805	*CNTFR*	A	G	411	REC	1.00524	0.514688	0.606771	ALL	0.343953	-0.278166	0.780885	0.265451	0.0886456	0.929364
9	34572767	rs10758268	*CNTFR*	A	C	411	REC	0.291109	-0.531789	0.594872	ALL	0.224128	-0.389564	0.696859	0.169787	0.544177	0.58632
9	87295237	rs1187350	*NTRK2*	G	A	411	REC	0.247789	-0.0860807	0.931402	ALL	0.224258	-0.394976	0.692861	0.167808	0.660799	0.508741
9	87295237	rs1187350	*NTRK2*	G	A	411	REC	0.247789	-0.0860807	0.931402	ALL	0.224258	-0.394976	0.692861	0.167808	0.660799	0.508741
9	87302196	rs1619120	*NTRK2*	A	G	407	REC	0.284268	-0.193282	0.846738	ALL	0.226442	-0.474181	0.635371	0.174041	1.35737	0.174663
9	87308783	rs1187343	*NTRK2*	G	A	410	REC	0.292153	0.0628063	0.949921	ALL	0.222048	-0.65469	0.512667	0.174141	1.75331	0.079549
9	87316037	rs1187337	*NTRK2*	A	G	411	REC	0.256916	0.361736	0.717549	ALL	0.22553	-0.981794	0.326201	0.170838	1.44973	0.147134
9	87355358	rs11140745	*NTRK2*	A	G	410	REC	0.311039	0.106569	0.915131	ALL	0.225797	-1.17575	0.239696	0.172107	0.0243066	0.980608
9	87387622	rs1573219	*NTRK2*	A	G	411	REC	0.332315	0.424579	0.671143	ALL	0.224408	-1.48111	0.138576	0.178862	0.668067	0.504091
9	87409025	rs1899640	*NTRK2*	G	A	411	REC	0.305216	0.158107	0.874373	ALL	0.222889	-0.918127	0.358553	0.170902	0.17145	0.86387
9	87414794	rs1187287	*NTRK2*	A	G	409	REC	0.549242	-0.537533	0.5909	ALL	0.298076	1.14374	0.252729	0.202139	-0.222902	0.823612
9	87415028	rs1187286	*NTRK2*	C	A	410	REC	0.396286	0.598382	0.549585	ALL	0.246037	-0.363113	0.71652	0.189052	0.350184	0.7262
9	87419117	rs716893	*NTRK2*	A	G	407	REC	0.300038	0.211226	0.832711	ALL	0.228281	-0.44552	0.655944	0.172307	-0.120311	0.904237
9	87421631	rs3739804	*NTRK2*	G	A	411	REC	0.766668	0.678327	0.497565	ALL	0.294415	-2.34926	0.0188106	0.274431	0.431286	0.666261
9	87441475	rs10512154	*NTRK2*	A	G	410	REC	0.515204	-1.03603	0.300186	ALL	0.299381	1.69708	0.0896816	0.197214	0.138381	0.88994
9	87447045	rs2083828	*NTRK2*	A	C	411	REC	0.240812	-1.06702	0.285962	ALL	0.22179	0.924519	0.355216	0.164459	-2.16E-15	1
9	87452058	rs7855888	*NTRK2*	G	A	411	REC	0.369497	0.389775	0.696703	ALL	0.245004	-0.00480505	0.996166	0.18189	-0.128313	0.897901
9	87473009	rs1838158	*NTRK2*	G	A	411	REC	0.546629	1.26076	0.207395	ALL	0.281072	-0.86803	0.385378	0.232024	0.282411	0.777628
9	87478135	rs7048015	*NTRK2*	C	A	411	REC	0.485693	0.623106	0.533215	ALL	0.258221	-0.852593	0.393885	0.207808	0.566406	0.571118
9	87491253	rs10780691	*NTRK2*	A	G	411	REC	0.246379	-0.780991	0.434808	ALL	0.224168	1.93354	0.0531696	0.161286	-0.327941	0.742956
9	87530935	rs10868238	*NTRK2*	A	G	410	REC	0.24839	-0.444708	0.656531	ALL	0.221675	-2.27391	0.0229714	0.164243	0.841291	0.400185
9	87551964	rs12340212	*NTRK2*	A	G	411	REC	1.07396	-1.35469	0.175516	ALL	0.288281	-1.31922	0.187096	0.229957	-0.403204	0.686798
9	87553563	rs6559838	*NTRK2*	A	G	411	REC	0.632215	-2.10779	0.0350491	ALL	0.251684	-1.26855	0.204603	0.201873	0.903506	0.366257
9	87558294	rs2808707	*NTRK2*	A	C	411	REC	0.250781	-0.0560784	0.955279	ALL	0.219696	-1.944	0.0518955	0.163387	-0.484862	0.627774
9	87575500	rs6559840	*NTRK2*	A	G	410	REC	0.448261	-1.03204	0.302054	ALL	0.271465	0.77288	0.439593	0.188425	-0.681668	0.495449
9	87585624	rs3860945	*NTRK2*	G	A	411	REC	0.797236	-1.11977	0.262811	ALL	0.321335	0.437289	0.661902	0.221949	-0.617476	0.536921
9	87590382	rs4877894	*NTRK2*	G	A	410	REC	0.261719	1.2672	0.205082	ALL	0.234822	-0.236837	0.812783	0.177311	-2.97264	0.002953
9	87593028	rs10868241	*NTRK2*	A	G	411	REC	0.383594	2.20403	0.0275224	ALL	0.251509	-0.619188	0.535792	0.184169	-2.44402	0.014525
9	87595734	rs4361832	*NTRK2*	A	G	411	REC	0.635303	1.73551	0.082651	ALL	0.291022	-0.266981	0.789484	0.209725	-1.51854	0.128878
9	87616257	rs1948308	*NTRK2*	G	A	411	REC	0.260961	0.0387621	0.96908	ALL	0.226115	-0.155424	0.876487	0.168067	-1.61863	0.105528
9	87616532	rs923559	*NTRK2*	A	G	411	REC	0.383678	0.14833	0.882082	ALL	0.249021	-0.37925	0.704502	0.179904	-2.0296	0.042397
9	87632993	rs1387924	*NTRK2*	A	C	411	REC	1.06562	-1.49436	0.13508	ALL	0.311952	0.357251	0.720904	0.219524	-0.666144	0.505319
11	27679916	rs6265	*BDNF*	A	G	411	REC	0.666336	-1.06833	0.285373	ALL	0.279712	0.356338	0.721587	0.238488	2.79041	0.005264

11	27679916	rs6265	*BDNF*	A	G	411	REC	0.666336	-1.06833	0.285373	ALL	0.279712	0.356338	0.721587	0.238488	2.79041	0.005264
11	27695910	rs10835210	*BDNF*	A	C	408	REC	0.273006	3.73657	0.0001865	ALL	0.227716	-1.78691	0.0739526	0.16938	-1.7379	0.082228
11	27701365	rs10835211	*BDNF*	A	G	411	REC	0.445149	-0.112683	0.910282	ALL	0.274274	0.888001	0.37454	0.196561	0.546876	0.584464
11	27720937	rs66866077	*BDNF*	A	G	411	REC	1.01594	-0.582426	0.56028	ALL	0.472102	0.271831	0.785752	0.324096	-0.201165	0.840569
11	27728539	rs2030323	*BDNF*	A	C	411	REC	0.555986	-1.38552	0.165893	ALL	0.261315	-0.078824	0.937173	0.222615	2.58923	0.009619
11	27731983	rs7934165	*BDNF*	A	G	411	REC	0.246757	4.37599	1.21E-05	ALL	0.229875	-1.59521	0.110664	0.172292	-2.32503	0.020071
11	58391501	rs1800169	*CNTF*	A	G	411	REC	0.621034	-0.300032	0.764153	ALL	0.318012	0.44145	0.658887	0.240321	0.840241	0.400774
12	5554678	rs10774329	*NTF3*	A	C	411	REC	0.592993	-0.871688	0.383379	ALL	0.282224	-0.109582	0.912741	0.208077	-0.414884	0.678227
12	5557893	rs10774330	*NTF3*	G	A	411	REC	0.310681	0.954084	0.340041	ALL	0.232968	-0.444581	0.656623	0.175159	-0.171569	0.863776
12	5575467	rs7974186	*NTF3*	A	G	411	REC	0.56596	1.2177	0.223337	ALL	0.285792	-0.251285	0.801594	0.21256	-0.456998	0.647672
12	5576594	rs7958038	*NTF3*	G	A	411	REC	0.393363	-0.0943597	0.924823	ALL	0.241546	-0.676509	0.498717	0.186127	0.0833882	0.933543
12	5587152	rs11063699	*NTF3*	C	A	411	REC	0.348368	-0.339481	0.734247	ALL	0.239488	-0.40857	0.682855	0.181291	0.258327	0.796155
12	5594403	rs11063708	*NTF3*	A	G	411	REC	0.249557	-0.241757	0.808968	ALL	0.223484	0.0620617	0.950514	0.166751	0.276902	0.781856
12	5603632	rs6332	*NTF3*	G	A	411	REC	0.246078	1.44316	0.148976	ALL	0.219423	-0.934005	0.350301	0.163996	-1.50892	0.131318
12	5613536	rs11612899	*NTF3*	A	G	411	REC	0.434518	0.00317218	0.997469	ALL	0.270017	0.677016	0.498396	0.19145	-0.007957	0.993652
12	5618814	rs11063723	*NTF3*	G	A	411	REC	0.26647	1.02051	0.307489	ALL	0.2236	-0.550476	0.581993	0.166877	-0.83867	0.401654
12	5626158	rs10774336	*NTF3*	A	G	408	REC	0.252701	-1.25615	0.209062	ALL	0.226806	-0.262621	0.792843	0.169273	1.5651	0.11756
12	5630281	rs10774339	*NTF3*	A	G	410	REC	0.265977	0.0356368	0.971572	ALL	0.222503	-1.01834	0.308519	0.16956	0.884262	0.376555
15	88419424	rs7176429	*NTRK3*	A	C	411	REC	0.299605	-0.594535	0.552154	ALL	0.222548	-0.559175	0.576042	0.172197	1.14088	0.253921
15	88423463	rs1560975	*NTRK3*	G	A	411	REC	0.264717	-1.19643	0.23153	ALL	0.216381	-0.809307	0.418338	0.16325	0.372922	0.709207
15	88428702	rs2117655	*NTRK3*	C	A	411	REC	0.263874	-0.749915	0.453306	ALL	0.212993	-0.463724	0.642845	0.161747	0.775183	0.438232
15	88430769	rs1369430	*NTRK3*	G	A	410	REC	0.282928	-0.534363	0.59309	ALL	0.21947	-1.1489	0.250598	0.166155	0.200239	0.841294
15	88454826	rs1435397	*NTRK3*	G	A	411	REC	0.468256	0.239583	0.810653	ALL	0.264968	0.120437	0.904137	0.204977	0.951848	0.341174
15	88463831	rs11855377	*NTRK3*	G	A	411	REC	0.250352	0.289525	0.77218	ALL	0.215689	-0.925505	0.354703	0.162058	0.236249	0.813239
15	88465057	rs1946697	*NTRK3*	A	C	411	REC	0.376247	0.916528	0.35939	ALL	0.235368	-1.43923	0.150085	0.183838	-0.530097	0.596045
15	88475356	rs8041239	*NTRK3*	G	A	410	REC	0.476281	0.441219	0.659055	ALL	0.27527	0.350812	0.725729	0.202944	0.500287	0.616873
15	88482578	rs8024898	*NTRK3*	A	G	411	REC	0.236995	-0.28755	0.773691	ALL	0.219493	1.55272	0.120491	0.160777	-0.092635	0.926194
15	88493008	rs8031871	*NTRK3*	A	G	411	REC	0.305327	0.837433	0.402349	ALL	0.22805	-1.32909	0.183818	0.173966	-0.321477	0.747849
15	88498466	rs11631508	*NTRK3*	G	A	411	REC	0.329953	-0.614241	0.539056	ALL	0.232024	0.326608	0.743965	0.173659	0.629138	0.529259
15	88499185	rs13380271	*NTRK3*	A	G	411	REC	0.26033	0.606991	0.543857	ALL	0.226651	-1.08048	0.279927	0.170071	0.101238	0.919361
15	88519216	rs2018052	*NTRK3*	A	G	411	REC	0.328267	0.429814	0.667331	ALL	0.238687	-0.00312032	0.99751	0.178407	0.127804	0.898304
15	88523321	rs11073755	*NTRK3*	G	A	411	REC	0.250679	-1.2572	0.208682	ALL	0.233898	1.61377	0.106577	0.171989	0.0990936	0.921064
15	88525951	rs16941103	*NTRK3*	A	G	411	REC	0.262332	-1.29099	0.196707	ALL	0.221826	-0.266976	0.789488	0.168626	1.73237	0.083207
15	88547290	rs8030107	*NTRK3*	A	G	411	REC	0.247822	-1.0228	0.3064	ALL	0.224979	0.449314	0.653205	0.168859	1.52259	0.127862
15	88584252	rs12594283	*NTRK3*	C	A	411	REC	0.371461	-1.24759	0.21218	ALL	0.241626	0.757902	0.44851	0.183828	1.70587	0.088033
15	88593449	rs4887350	*NTRK3*	G	A	407	REC	0.431409	-1.04045	0.298131	ALL	0.255197	0.518045	0.604427	0.190014	0.720821	0.47102
15	88602841	rs3825885	*NTRK3*	G	A	411	REC	0.375548	-0.652703	0.513948	ALL	0.260429	1.41889	0.155932	0.181124	0.0686683	0.945254
15	88658964	rs11636250	*NTRK3*	G	A	411	REC	0.450161	-0.273853	0.784198	ALL	0.274071	0.961923	0.336088	0.190793	-0.3007	0.763643
15	88661739	rs9806762	*NTRK3*	G	A	411	REC	0.376446	-1.99787	0.0457307	ALL	0.253835	1.98159	0.0475248	0.181392	1.56834	0.116801
15	88665139	rs2349057	*NTRK3*	G	A	411	REC	0.474556	-0.401466	0.688077	ALL	0.2794	1.104	0.269594	0.192905	-0.551862	0.581043
15	88671372	rs1104765	*NTRK3*	A	C	411	REC	1.02993	-2.48377	0.0129999	ALL	0.277522	1.5217	0.128084	0.208548	2.39896	0.016442
15	88674990	rs8035239	*NTRK3*	A	G	411	REC	0.258999	-1.89239	0.0584391	ALL	0.227196	2.12601	0.0335023	0.165677	1.53658	0.124395
15	88676679	rs4887368	*NTRK3*	A	G	411	REC	0.565136	-1.07582	0.282008	ALL	0.287387	0.898472	0.368934	0.205758	0.511444	0.60904
15	88688097	rs3784404	*NTRK3*	A	G	411	REC	0.278446	-1.78849	0.0736971	ALL	0.228115	1.66643	0.0956276	0.168098	1.80889	0.070468

15	88699342	rs16941334	*NTRK3*	A	C	411	REC	0.714886	-2.16796	0.0301619	ALL	0.277732	0.532163	0.594613	0.211301	1.20222	0.229279
15	88703546	rs12148845	*NTRK3*	G	A	411	REC	0.707166	-2.54947	0.0107888	ALL	0.278062	1.29349	0.195841	0.204249	1.65821	0.097275
15	88706936	rs11073767	*NTRK3*	A	C	411	REC	0.269694	-1.71212	0.0868742	ALL	0.225671	1.50624	0.132007	0.166748	1.62123	0.104969
15	88717708	rs6496466	*NTRK3*	G	A	411	REC	0.498966	-0.704354	0.481213	ALL	0.281912	1.03953	0.298558	0.198684	0.441376	0.658941
15	88735310	rs6496469	*NTRK3*	G	A	411	REC	0.494604	0.1272	0.898782	ALL	0.275091	0.434657	0.663811	0.20234	0.362147	0.717242
15	88737328	rs4887381	*NTRK3*	A	C	411	REC	0.622661	-2.56775	0.010236	ALL	0.280724	1.66734	0.0954475	0.19503	1.33295	0.182548
15	88737834	rs8025146	*NTRK3*	A	C	411	REC	0.416645	0.107957	0.91403	ALL	0.271518	1.25952	0.207844	0.188911	0.272189	0.785477
15	88758621	rs1107292	*NTRK3*	G	C	411	REC	1.15923	-0.510441	0.609743	ALL	0.322729	0.170201	0.864852	0.251103	1.06403	0.287317
15	88771571	rs11635443	*NTRK3*	A	G	411	REC	1.08498	-1.19518	0.232018	ALL	0.390127	1.7526	0.0796705	0.271682	2.06399	0.039019
15	88790325	rs4887399	*NTRK3*	C	A	411	REC	1.10023	-1.00929	0.312837	ALL	0.37666	1.60902	0.107612	0.254059	1.35331	0.175957
15	88794859	rs4887400	*NTRK3*	C	A	411	REC	0.377111	-0.893869	0.371392	ALL	0.249516	0.54148	0.588177	0.176381	-1.05699	0.290516
17	47578118	rs3785931	*NGFR*	G	A	405	REC	0.346893	0.335134	0.737524	ALL	0.246578	0.585953	0.557907	0.176247	-0.470332	0.638118
17	47587819	rs2072446	*NGFR*	A	G	411	REC	NA	NA	NA	ALL	0.740252	1.82218	0.0684275	0.299129	-1.11359	0.265457
