# Supplementary material for: Clustering suicidal phenotypes and genetic associations with brain-derived neurotrophic factor in patients with substance use disorders
Source: Transl Psychiatry. 2021 Jan 21;11:72. doi: 10.1038/s41398-021-01200-5 (PMC7820499; doi:10.1038/s41398-021-01200-5)
Supplement: Supplementary file 3 — Supplementary Table 5B [file 41398_2021_1200_MOESM3_ESM.pdf]

Supplementary Table 5B: summary statistics of genetic associations between the BDNF pathway and recurrent suicide attempts.

							Recurrent SA (multinomial regression with no SA as the reference variable)						
								Exactly one SA			*≥ 2 SAs*		
CHROM	POS	rsID	gene	A1	A2	N	TEST	SE	Z	P	SE	Z	P
1	109856306	rs464218	*SORT1*	G	A	411	ALL	0.21605	0.04264	0.965989	0.17558	-1.95631	0.0504291
1	109880721	rs11102972	*SORT1*	G	A	411	ALL	0.25875	0.494307	0.621089	0.19698	-1.0106	0.312208
1	109923677	rs12037569	*SORT1*	A	C	411	ALL	0.27697	-1.28368	0.199253	0.24555	0.0027821	0.99778
1	156785617	rs1800601	*NTRK1*	G	A	411	ALL	0.24412	2.51857	0.011783	0.17916	0.338967	0.734635
1	156796697	rs7522395	*NTRK1*	A	G	411	ALL	0.27149	1.88734	0.059115	0.19348	0.236911	0.812726
1	156802657	rs4661061	*NTRK1*	A	G	411	ALL	0.24539	1.70172	0.088807	0.18905	1.07416	0.282751
1	156805803	rs7534418	*NTRK1*	A	G	411	ALL	0.22864	1.58858	0.112156	0.17616	0.349889	0.726422
9	34554999	rs7044318	*CNTFR*	A	G	411	ALL	0.2691	0.136244	0.891629	0.21417	-0.456743	0.647856
9	34564274	rs4879805	*CNTFR*	A	G	411	ALL	0.33115	-0.0370152	0.970473	0.27026	-0.066035	0.94735
9	34572767	rs10758268	*CNTFR*	A	C	411	ALL	0.21251	0.0236792	0.981109	0.17398	0.168772	0.865976
9	87295237	rs1187350	*NTRK2*	G	A	411	ALL	0.21063	-0.0758111	0.939569	0.17249	0.487648	0.625799
9	87295237	rs1187350	*NTRK2*	G	A	411	ALL	0.21063	-0.0758111	0.939569	0.17249	0.487648	0.625799
9	87302196	rs1619120	*NTRK2*	A	G	407	ALL	0.2155	0.0527993	0.957892	0.17747	1.06403	0.287315
9	87308783	rs1187343	*NTRK2*	G	A	410	ALL	0.21443	0.629807	0.528821	0.17515	0.818351	0.413157
9	87316037	rs1187337	*NTRK2*	A	G	411	ALL	0.21199	0.194179	0.846036	0.17391	0.728371	0.466387
9	87355358	rs11140745	*NTRK2*	A	G	410	ALL	0.21238	-1.01698	0.309164	0.17716	0.112727	0.910247
9	87387622	rs1573219	*NTRK2*	A	G	411	ALL	0.21295	-1.04104	0.297856	0.18337	0.596204	0.551039
9	87409025	rs1899640	*NTRK2*	G	A	411	ALL	0.20927	-0.94002	0.347207	0.17657	0.382742	0.701911
9	87414794	rs1187287	*NTRK2*	A	G	409	ALL	0.2576	0.0765217	0.939004	0.21395	0.465359	0.641674
9	87415028	rs1187286	*NTRK2*	C	A	410	ALL	0.23838	0.268216	0.788533	0.19173	-0.069822	0.944336
9	87419117	rs716893	*NTRK2*	A	G	407	ALL	0.21709	0.0912834	0.927267	0.17643	-0.490746	0.623606
9	87421631	rs3739804	*NTRK2*	G	A	411	ALL	0.34612	0.329172	0.742026	0.24845	-1.40753	0.159269
9	87441475	rs10512154	*NTRK2*	A	G	410	ALL	0.2547	0.598583	0.549451	0.20917	0.832233	0.405277
9	87447045	rs2083828	*NTRK2*	A	C	411	ALL	0.20713	0.169493	0.865409	0.16936	0.492566	0.622319
9	87452058	rs7855888	*NTRK2*	G	A	411	ALL	0.24153	0.771348	0.440501	0.18352	-0.822188	0.41097
9	87473009	rs1838158	*NTRK2*	G	A	411	ALL	0.2988	0.412071	0.680288	0.22691	-0.416342	0.67716
9	87478135	rs7048015	*NTRK2*	C	A	411	ALL	0.25318	-0.102085	0.918689	0.20901	0.0903534	0.928006

9	87491253	rs10780691	*NTRK2*	A	G	411	ALL	0.20294	0.0562166	0.955169	0.16677	0.907137	0.364335
9	87530935	rs10868238	*NTRK2*	A	G	410	ALL	0.20515	0.277234	0.781601	0.16679	-0.848576	0.396117
9	87551964	rs12340212	*NTRK2*	A	G	411	ALL	0.28698	-0.408104	0.683197	0.23002	-1.00695	0.313961
9	87553563	rs6559838	*NTRK2*	A	G	411	ALL	0.25678	0.867142	0.385864	0.199	-0.392582	0.694628
9	87558294	rs2808707	*NTRK2*	A	C	411	ALL	0.20539	-0.465648	0.641467	0.1678	-1.46985	0.141603
9	87575500	rs6559840	*NTRK2*	A	G	410	ALL	0.24215	0.0763493	0.939141	0.19422	-0.409544	0.68214
9	87585624	rs3860945	*NTRK2*	G	A	411	ALL	0.28378	-0.217081	0.828145	0.23303	-0.211528	0.832476
9	87590382	rs4877894	*NTRK2*	G	A	410	ALL	0.21915	-1.17369	0.240519	0.1811	-2.45182	0.0142136
9	87593028	rs10868241	*NTRK2*	A	G	411	ALL	0.23462	-0.904646	0.365653	0.18924	-2.33713	0.0194325
9	87595734	rs4361832	*NTRK2*	A	G	411	ALL	0.26513	-0.931629	0.351528	0.21703	-1.27447	0.202499
9	87616257	rs1948308	*NTRK2*	G	A	411	ALL	0.21048	-1.0522	0.292709	0.17227	-1.16797	0.242817
9	87616532	rs923559	*NTRK2*	A	G	411	ALL	0.22747	-1.12019	0.262631	0.18607	-1.5962	0.110443
9	87632993	rs1387924	*NTRK2*	A	C	411	ALL	0.27335	-0.67452	0.49998	0.23113	-0.147979	0.88236
11	27679916	rs6265	*BDNF*	A	G	411	ALL	0.28486	1.50087	0.13339	0.23143	1.90327	0.0570048
11	27679916	rs6265	*BDNF*	A	G	411	ALL	0.28486	1.50087	0.13339	0.23143	1.90327	0.0570048
11	27695910	rs1083521(	*BDNF*	A	C	408	ALL	0.21289	-1.47164	0.141118	0.17532	-2.078	0.0377092
11	27701365	rs10835211	*BDNF*	A	G	411	ALL	0.23951	-0.23013	0.81799	0.21129	1.34767	0.177766
11	27720937	rs66866077	*BDNF*	A	G	411	ALL	0.52343	0.933798	0.350408	0.31669	-0.671493	0.501907
11	27728539	rs2030323	*BDNF*	A	C	411	ALL	0.27147	1.52652	0.126881	0.21376	1.38492	0.166078
11	27731983	rs7934165	*BDNF*	A	G	411	ALL	0.21602	-1.09598	0.273089	0.17928	-2.83752	0.00454656
11	58391501	rs1800169	*CNTF*	A	G	411	ALL	0.28387	-0.0072364	0.994226	0.25309	1.02013	0.307668
12	5554678	rs1077432(	*NTF3*	A	C	411	ALL	0.26488	-0.12999	0.896575	0.21394	-0.415313	0.677913
12	5557893	rs1077433(	*NTF3*	G	A	411	ALL	0.21962	-0.31511	0.752678	0.18001	-0.247453	0.804557
12	5575467	rs7974186	*NTF3*	A	G	411	ALL	0.29439	0.848172	0.396342	0.21203	-1.22952	0.218878
12	5576594	rs7958038	*NTF3*	G	A	411	ALL	0.22536	-0.956848	0.338644	0.19346	0.339247	0.734424
12	5587152	rs1106369(	*NTF3*	C	A	411	ALL	0.22301	-1.22488	0.220619	0.18945	0.766046	0.443649
12	5594403	rs1106370(	*NTF3*	A	G	411	ALL	0.21026	-0.534187	0.593212	0.17248	0.597202	0.550373
12	5603632	rs6332	*NTF3*	G	A	411	ALL	0.20564	-1.2134	0.224978	0.1683	-1.44433	0.148646
12	5613536	rs1161289(	*NTF3*	A	G	411	ALL	0.23583	-0.446652	0.655126	0.20345	0.628477	0.529692
12	5618814	rs1106372(	*NTF3*	G	A	411	ALL	0.20984	-0.436979	0.662127	0.17101	-1.05133	0.293106

12	5626158	rs10774336	*NTF3*	A	G	408	ALL	0.21312	1.29817	0.194229	0.17334	0.536438	0.591656
12	5630281	rs10774339	*NTF3*	A	G	410	ALL	0.21782	1.44305	0.149006	0.17232	-0.933734	0.350441
15	88419424	rs7176429	*NTRK3*	A	C	411	ALL	0.20885	-0.704043	0.481406	0.17861	1.34327	0.179183
15	88423463	rs1560975	*NTRK3*	G	A	411	ALL	0.2033	-0.942931	0.345716	0.1685	0.540962	0.588534
15	88428702	rs2117655	*NTRK3*	C	A	411	ALL	0.19986	-0.654384	0.512864	0.16735	0.984936	0.324656
15	88430769	rs1369430	*NTRK3*	G	A	410	ALL	0.20455	-1.25403	0.209832	0.17234	0.372227	0.709724
15	88454826	rs1435397	*NTRK3*	G	A	411	ALL	0.24747	0.0126026	0.989945	0.21148	0.925465	0.354724
15	88463831	rs11855377	*NTRK3*	G	A	411	ALL	0.20388	-1.51051	0.130912	0.16883	0.858884	0.390404
15	88465057	rs1946697	*NTRK3*	A	C	411	ALL	0.2235	-1.33741	0.181088	0.1912	-0.270809	0.786538
15	88475356	rs8041239	*NTRK3*	G	A	410	ALL	0.23979	-1.0267	0.30456	0.2208	1.38188	0.167009
15	88482578	rs8024898	*NTRK3*	A	G	411	ALL	0.20715	1.74478	0.081023	0.16607	-0.452613	0.650827
15	88493008	rs8031871	*NTRK3*	A	G	411	ALL	0.21475	-1.4987	0.133952	0.18098	0.131019	0.89576
15	88498466	rs11631508	*NTRK3*	G	A	411	ALL	0.20922	-0.923333	0.355834	0.18608	1.57305	0.115707
15	88499185	rs13380271	*NTRK3*	A	G	411	ALL	0.21303	-1.17845	0.238616	0.17533	0.245479	0.806086
15	88519216	rs2018052	*NTRK3*	A	G	411	ALL	0.22543	0.251224	0.801641	0.18315	0.082924	0.933912
15	88523321	rs11073755	*NTRK3*	G	A	411	ALL	0.21644	0.25564	0.798229	0.17736	0.995434	0.319525
15	88525951	rs16941103	*NTRK3*	A	G	411	ALL	0.20825	-0.510185	0.609922	0.17382	1.76794	0.0770701
15	88547290	rs8030107	*NTRK3*	A	G	411	ALL	0.21063	0.248776	0.803534	0.17357	1.59017	0.111797
15	88584252	rs12594283	*NTRK3*	C	A	411	ALL	0.21953	-0.173382	0.862351	0.19554	2.3071	0.0210492
15	88593449	rs4887350	*NTRK3*	G	A	407	ALL	0.22914	-0.928927	0.352927	0.20365	1.66234	0.0964437
15	88602841	rs3825885	*NTRK3*	G	A	411	ALL	0.2204	-1.68728	0.09155	0.20343	2.29567	0.0216945
15	88658964	rs11636250	*NTRK3*	G	A	411	ALL	0.25178	0.677769	0.497918	0.19645	-0.314645	0.753031
15	88661739	rs9806762	*NTRK3*	G	A	411	ALL	0.22748	1.07204	0.2837	0.19145	2.32181	0.020243
15	88665139	rs2349057	*NTRK3*	G	A	411	ALL	0.25137	0.416262	0.677218	0.19981	-0.302109	0.762569
15	88671372	rs1104765	*NTRK3*	A	C	411	ALL	0.24694	0.688752	0.490979	0.225	3.04367	0.00233707
15	88674990	rs8035239	*NTRK3*	A	G	411	ALL	0.20762	0.770401	0.441062	0.17331	2.47027	0.013501
15	88676679	rs4887368	*NTRK3*	A	G	411	ALL	0.25885	0.314626	0.753046	0.21464	0.757418	0.4488
15	88688097	rs3784404	*NTRK3*	A	G	411	ALL	0.2077	0.209315	0.834202	0.17847	2.85571	0.00429401
15	88699342	rs16941334	*NTRK3*	A	C	411	ALL	0.24581	-0.442055	0.65845	0.23239	2.12002	0.0340041
15	88703546	rs12148845	*NTRK3*	G	A	411	ALL	0.24285	0.207944	0.835273	0.22431	2.61482	0.00892741

15	88706936	rs11073767	*NTRK3*	A	C	411	ALL	0.20746	0.433779	0.664449	0.17485	2.39071	0.016816
15	88717708	rs6496466	*NTRK3*	G	A	411	ALL	0.26041	0.892959	0.371879	0.20424	0.360939	0.718145
15	88735310	rs6496469	*NTRK3*	G	A	411	ALL	0.26717	0.936599	0.348965	0.20449	-0.155811	0.876182
15	88737328	rs4887381	*NTRK3*	A	C	411	ALL	0.2424	0.774112	0.438865	0.21097	2.09938	0.0357833
15	88737834	rs8025146	*NTRK3*	A	C	411	ALL	0.25208	1.1995	0.230335	0.19438	0.253525	0.799862
15	88758621	rs1107292	*NTRK3*	G	C	411	ALL	0.31744	0.788649	0.430317	0.25058	0.472425	0.636624
15	88771571	rs11635443	*NTRK3*	A	G	411	ALL	0.41113	2.4109	0.015913	0.26744	1.55492	0.119964
15	88790325	rs4887399	*NTRK3*	C	A	411	ALL	0.35885	1.83354	0.066723	0.25874	1.15939	0.246298
15	88794859	rs4887400	*NTRK3*	C	A	411	ALL	0.22591	-0.156168	0.8759	0.18308	-0.515567	0.606157
17	47578118	rs3785931	*NGFR*	G	A	405	ALL	0.222	-0.278403	0.780703	0.18418	0.0921872	0.926549
17	47587819	rs2072446	*NGFR*	A	G	411	ALL	0.35291	-1.52206	0.127994	0.37365	0.959593	0.33726
