# Supplementary material for: Clustering suicidal phenotypes and genetic associations with brain-derived neurotrophic factor in patients with substance use disorders
Source: Transl Psychiatry. 2021 Jan 21;11:72. doi: 10.1038/s41398-021-01200-5 (PMC7820499; doi:10.1038/s41398-021-01200-5)
Supplement: Supplementary file 4 — Supplementary Table 6 [file 41398_2021_1200_MOESM4_ESM.pdf]

Supplementary Table 6: summary statistics of 405 significant genetic associations between the clinical (“cases”) sample and controls regarding SA “not specified”.

Chromosome	SNPid	corrected *p*-value
1	rs10909901	0.000999
1	rs6424069	0.000999
1	rs301798	0.02198
1	rs4908760	0.000999
1	rs4908501	0.002997
1	rs219002	0.001998
1	rs837398	0.000999
1	rs2297812	0.000999
1	rs554870	0.001998
1	rs2803136	0.005994
1	rs1937791	0.000999
1	rs6593602	0.000999
1	rs3754112	0.04396
1	rs7526517	0.000999
1	rs3767333	0.01998
1	rs16835564	0.000999
1	rs6428233	0.005994
1	rs10494841	0.001998
1	rs12239515	0.03097
1	rs823066	0.007992
1	rs1800896	0.01598
1	rs7538655	0.002997
1	rs10779265	0.000999
1	rs945717	0.007992
1	rs1153952	0.02398
1	rs1153947	0.002997
1	rs801109	0.001998
1	rs1341715	0.007992
1	rs12086015	0.04595
1	rs6429449	0.004995
1	rs4658622	0.001998
2	rs6732271	0.004995
2	rs2347857	0.01299
2	rs10189542	0.000999
2	rs7606055	0.01598
2	rs1056836	0.000999
2	rs1370637	0.02597
2	rs7594227	0.000999
2	rs10179966	0.02997
2	rs700851	0.002997
2	rs6541806	0.00999
2	rs4241113	0.02098
2	rs11692586	0.04196
2	rs935471	0.000999
2	rs7607690	0.008991
2	rs935480	0.00999
2	rs755503	0.000999

2	rs515983	0.000999
2	rs6750788	0.000999
2	rs6705916	0.000999
2	rs10164986	0.000999
2	rs6747870	0.000999
2	rs6723108	0.000999
2	rs17698151	0.000999
2	rs6430539	0.000999
2	rs6714498	0.000999
2	rs10496732	0.000999
2	rs2874714	0.000999
2	rs16831235	0.03397
2	rs16831243	0.000999
2	rs7582173	0.000999
2	rs4954218	0.000999
2	rs7570971	0.000999
2	rs10445686	0.000999
2	rs3806502	0.01598
2	rs961360	0.000999
2	rs1446585	0.000999
2	rs1438307	0.000999
2	rs6430585	0.000999
2	rs10188066	0.000999
2	rs2322659	0.000999
2	rs3769013	0.000999
2	rs3754689	0.000999
2	rs12472293	0.000999
2	rs309152	0.000999
2	rs749873	0.000999
2	rs953387	0.000999
2	rs6430612	0.000999
2	rs4477975	0.005994
2	rs4954592	0.005994
2	rs11890854	0.000999
2	rs10197003	0.004995
2	rs635018	0.001998
2	rs578935	0.001998
2	rs47150	0.000999
2	rs1374111	0.005994
2	rs1593759	0.001998
2	rs778211	0.001998
2	rs16843069	0.02298
2	rs12691813	0.02997
2	rs1871780	0.000999
2	rs3821290	0.03896
2	rs11757	0.000999
2	rs4404233	0.02997
2	rs6735981	0.02098

2	rs1012337	0.01998
2	rs897476	0.01698
2	rs959427	0.01698
2	rs893451	0.02398
2	rs2720100	0.04895
2	rs6728476	0.03896
3	rs12495328	0.02198
3	rs970930	0.01998
3	rs1392702	0.004995
3	rs1512522	0.008991
3	rs5013525	0.000999
3	rs2117999	0.01698
3	rs3851366	0.01698
3	rs16866197	0.005994
4	rs9328764	0.007992
4	rs6830513	0.006993
4	rs10011549	0.007992
4	rs2236998	0.002997
4	rs12500979	0.04196
4	rs6826089	0.008991
4	rs13121107	0.03497
4	rs885395	0.002997
4	rs4833095	0.000999
4	rs1993585	0.007992
4	rs1486493	0.005994
4	rs3733553	0.001998
4	rs10516428	0.007992
4	rs4147541	0.000999
4	rs9884929	0.02997
4	rs12508528	0.001998
4	rs7655676	0.03896
4	rs4690859	0.01698
4	rs2321377	0.02797
4	rs6553418	0.01499
4	rs9312489	0.03097
4	rs3114031	0.03097
4	rs7689898	0.01698
5	rs4147775	0.000999
5	rs12109816	0.02797
5	rs6450233	0.00999
5	rs164572	0.003996
5	rs1042714	0.02398
5	rs1030206	0.01399
5	rs10073580	0.007992
5	rs2973775	0.02298
6	rs2070998	0.000999
6	rs7775656	0.003996
6	rs2057184	0.007992

6	rs1205852	0.001998
6	rs4142551	0.005994
6	rs2228375	0.001998
6	rs12199222	0.007992
6	rs6456433	0.004995
6	rs6918101	0.008991
6	rs3749971	0.01898
6	rs1235162	0.01898
6	rs8321	0.01199
6	rs9261290	0.01598
6	rs2523989	0.03197
6	rs2523987	0.005994
6	rs3130380	0.005994
6	rs3132610	0.00999
6	rs3094097	0.001998
6	rs9262143	0.003996
6	rs3130564	0.001998
6	rs1131896	0.000999
6	rs2256028	0.000999
6	rs3128982	0.01399
6	rs12665745	0.00999
6	rs2844463	0.001998
6	rs644827	0.000999
6	rs2242665	0.001998
6	rs2734335	0.008991
6	rs630379	0.000999
6	rs592229	0.000999
6	rs389883	0.000999
6	rs12333245	0.000999
6	rs2269429	0.000999
6	rs185819	0.03197
6	rs1150754	0.01399
6	rs169496	0.000999
6	rs204899	0.000999
6	rs408359	0.04396
6	rs204993	0.03097
6	rs415929	0.04795
6	rs2239804	0.007992
6	rs2858331	0.000999
6	rs513349	0.003996
6	rs2076173	0.000999
6	rs259686	0.001998
6	rs1574430	0.005994
6	rs2242416	0.007992
6	rs2487663	0.01898
6	rs12192544	0.000999
6	rs3963	0.01898
6	rs9454257	0.01399

6	rs7760466	0.02498
6	rs3793048	0.000999
6	rs240399	0.04196
6	rs9362001	0.02398
6	rs9388472	0.02697
6	rs6922771	0.01499
6	rs17304375	0.03896
6	rs1335295	0.02098
6	rs7772605	0.03996
6	rs901363	0.01998
6	rs753017	0.000999
6	rs2076828	0.000999
6	rs12526681	0.02098
6	rs2881194	0.02797
6	rs7747434	0.01499
7	rs1881116	0.03197
7	rs6975043	0.01598
7	rs1437490	0.04296
7	rs6955922	0.005994
7	rs6951573	0.01698
7	rs6958292	0.008991
7	rs6978592	0.01399
7	rs6959354	0.01598
7	rs10155882	0.007992
7	rs4148686	0.000999
7	rs2590634	0.01499
7	rs1006091	0.01499
7	rs10435284	0.01698
7	rs4731367	0.04196
7	rs6974804	0.00999
7	rs1222430	0.04595
7	rs194150	0.000999
7	rs965003	0.001998
7	rs7796764	0.03996
8	rs9785100	0.02398
8	rs11204117	0.01199
8	rs4921720	0.02398
8	rs2613675	0.04595
8	rs989329	0.01998
8	rs2631864	0.004995
8	rs519676	0.002997
8	rs511544	0.000999
8	rs2977016	0.007992
8	rs166928	0.04096
8	rs284818	0.02398
8	rs1531658	0.001998
8	rs6989572	0.007992
8	rs2555588	0.000999

8	rs7818862	0.02797
8	rs1879199	0.02398
8	rs16880099	0.001998
8	rs7826477	0.01598
8	rs2071598	0.02797
8	rs10081435	0.04795
8	rs1004380	0.007992
9	rs10814219	0.001998
9	rs13287364	0.02398
9	rs683	0.04296
9	rs2153271	0.03097
9	rs1538590	0.008991
9	rs1330327	0.00999
9	rs4745072	0.008991
9	rs7022575	0.007992
9	rs7037280	0.000999
9	rs4979136	0.002997
9	rs10817694	0.000999
9	rs10818288	0.001998
9	rs10818930	0.01898
9	rs7872110	0.01399
10	rs3995590	0.00999
10	rs1218471	0.005994
10	rs7069690	0.02697
10	rs4414112	0.02398
10	rs1871452	0.000999
10	rs4934207	0.007992
10	rs2270962	0.007992
10	rs17113829	0.04296
10	rs1033772	0.02997
11	rs4341514	0.01399
11	rs4320932	0.004995
11	rs11028621	0.01399
11	rs7939568	0.007992
11	rs932287	0.02098
11	rs7926443	0.01698
11	rs3802967	0.000999
11	rs2922049	0.008991
11	rs4755844	0.001998
11	rs7130768	0.01399
11	rs7125035	0.005994
11	rs11233471	0.007992
11	rs10830989	0.000999
11	rs7943420	0.01399
11	rs1938623	0.004995
12	rs527118	0.00999
12	rs526282	0.000999
12	rs735295	0.02398

12	rs3741920	0.000999
12	rs2040352	0.04296
12	rs7304507	0.000999
12	rs3741841	0.01998
12	rs2216228	0.02398
12	rs12817074	0.008991
12	rs2196488	0.007992
12	rs7486863	0.005994
12	rs10748036	0.04595
12	rs7301453	0.004995
12	rs559392	0.02198
12	rs2008370	0.005994
12	rs10850577	0.007992
12	rs12227287	0.02797
12	rs11057830	0.002997
13	rs7984141	0.03397
13	rs7333322	0.001998
13	rs3783036	0.03896
13	rs1407961	0.000999
13	rs9509028	0.003996
13	rs4769541	0.03097
13	rs4769852	0.007992
13	rs3742302	0.01598
13	rs9594751	0.04196
13	rs9588629	0.005994
13	rs4773558	0.000999
13	rs9584410	0.008991
13	rs279942	0.000999
13	rs1330518	0.004995
13	rs2257298	0.04795
14	rs4981439	0.002997
14	rs2273394	0.000999
14	rs12879346	0.000999
14	rs1884777	0.01998
14	rs2038281	0.005994
14	rs8004890	0.03097
14	rs2244057	0.02098
14	rs12434438	0.000999
14	rs9944035	0.01499
14	rs1570295	0.01199
14	rs1269068	0.03696
14	rs12889741	0.01598
14	rs1424847	0.04595
14	rs3844534	0.007992
14	rs10134510	0.000999
14	rs2039478	0.000999
14	rs4904868	0.02697
14	rs2069590	0.000999

14	rs3809470	0.01598
14	rs4983579	0.008991
15	rs10873631	0.005994
15	rs4600441	0.03097
15	rs6497238	0.01399
15	rs4778192	0.01199
15	rs8035334	0.00999
15	rs11852452	0.008991
15	rs4778232	0.001998
15	rs16950821	0.01399
15	rs1597196	0.01998
15	rs7174027	0.000999
15	rs4778138	0.000999
15	rs7495174	0.000999
15	rs12913832	0.000999
15	rs1667394	0.000999
15	rs1545471	0.01399
15	rs17649643	0.008991
16	rs6500949	0.03996
16	rs4781504	0.01698
16	rs12598337	0.00999
16	rs7204509	0.002997
16	rs4781928	0.00999
16	rs1004101	0.000999
16	rs12325563	0.007992
16	rs8050354	0.03197
16	rs9934800	0.00999
16	rs7188406	0.02398
16	rs3748400	0.04096
16	rs1060253	0.000999
16	rs3743975	0.01399
16	rs889574	0.04595
16	rs2911253	0.001998
16	rs2965946	0.003996
16	rs258322	0.008991
17	rs9905797	0.03996
17	rs931908	0.000999
17	rs7946	0.000999
17	rs2228100	0.000999
17	rs4796093	0.01199
17	rs1558748	0.02897
17	rs718073	0.02498
17	rs9901648	0.01399
18	rs12608027	0.00999
18	rs1441002	0.01399
18	rs499468	0.005994
18	rs1543073	0.04595
18	rs7240273	0.01499

18	rs7244157	0.04396
18	rs309251	0.005994
19	rs8289	0.007992
19	rs7246083	0.007992
19	rs731773	0.01898
19	rs714772	0.000999
19	rs2239367	0.000999
19	rs2082455	0.004995
19	rs4806152	0.02797
19	rs17305311	0.01399
20	rs619865	0.03097
20	rs7271624	0.000999
20	rs2235617	0.000999
20	rs6024282	0.005994
20	rs2248900	0.03996
21	rs8133689	0.001998
21	rs728015	0.004995
21	rs2824444	0.004995
21	rs2835006	0.03097
21	rs425215	0.000999
22	rs132028	0.004995
22	rs6520069	0.01199
