# Supplementary material for: Clustering suicidal phenotypes and genetic associations with brain-derived neurotrophic factor in patients with substance use disorders
Source: Transl Psychiatry. 2021 Jan 21;11:72. doi: 10.1038/s41398-021-01200-5 (PMC7820499; doi:10.1038/s41398-021-01200-5)
Supplement: Supplementary file 6 — Supplementary Figure 1 [file 41398_2021_1200_MOESM6_ESM.docx]

Supplementary Figure 1: regional plot for associations between SNPs in the *BDNF* gene and lifetime SA.

rs10835210
