# Supplementary material for: Clustering suicidal phenotypes and genetic associations with brain-derived neurotrophic factor in patients with substance use disorders
Source: Transl Psychiatry. 2021 Jan 21;11:72. doi: 10.1038/s41398-021-01200-5 (PMC7820499; doi:10.1038/s41398-021-01200-5)
Supplement: Supplementary file 7 — Supplementary Tables 2 to 4 [file 41398_2021_1200_MOESM7_ESM.pdf]

Supplementary tables 2 to 4

	**No lifetime SA, N=287 (63%)**	**Lifetime SA, N=169 (37%)**	**Odds ratio (OR) [95% confidence interval]**	***p*-value (Chi² or Mann-Whitney)**	**N**
Cigarettes per day	16 (9)	21 (11)	1.05 [1.03;1.07]	***<0.001****	411
Sedative use disorder	122 (46%)	112 (67%)	2.43 [1.63;3.65]	***<0.001****	435
Cocaine use disorder	248 (91%)	148 (92%)	1.19 [0.6;2.46]	0.745	435
Alcohol use disorder	191 (71%)	126 (78%)	1.48 [0.94;2.37]	0.11	431
Cannabis use disorder	215 (79%)	136 (83%)	1.28 [0.78;2.15]	0.386	436
Opiates use disorder	204 (72%)	124 (73%)	1.09 [0.71;1.69]	0.761	454
Number of SUDs ≥3	117 (40.8%)	94 (55.6%)	1.82 [1.24;2.68]	***0.002****	456
Gender (Women vs. Men)	51 (18%)	52 (31%)	2.05 [1.31;3.21]	***0.002****	456
Age	39 (8)	39 (10)	1 [0.98;1.02]	0.849	456
Homelessness ≥ 3 months	64 (26%)	53 (35%)	1.53 [0.98;2.37]	0.073	399
Number of current medications	2.44 (1.98)	2.96 (1.88)	1.15 [1.04;1.27]	***0.006****	456
Current antidepressant treatment	64 (22%)	49 (29%)	1.42 [0.92;2.19]	0.137	456
Current number of psychotropic medications	1.16 (1.08)	1.61 (1.14)	1.44 [1.21;1.71]	***<0.001****	456
Current number of non-psychotropic medications	1.29 (1.42)	1.36 (1.29)	1.04 [0.9;1.19]	0.602	456
Protocol of origin (#2 vs. #1)	187 (65%)	117 (69%)	1.2 [0.8;1.81]	0.43	456

Supplementary Table 2: sample description and bivariate associations with lifetime suicide attempt (SA). Data are presented as median (interquartile range) or N (%). ***p* <0.05 after Bonferroni correction for three tests**.

	No lifetime SA, N=287 (63%)	At least one, but not serious SA, N=54 (12%)	At least one serious SA, N =114 (25%)	*p*-value (Chi^2 or Kruskal-Wallis)				N
				overall	No SA *vs*. at least 1 non-serious SA	No SA *vs*. at least one serious SA	1 non-serious *vs*. at least one serious SA	
One SA	0 (0%)	23 (43%)	43 (38%)	<0.001*[a]	<0.001*	<0.001*	0.614	454
≥2 SAs	0 (0%)	31 (57%)	70 (62%)					
Cigarettes per day	15 [10;20]	20 [10;26]	20 [15;25]	<0.001*[a]	0.007*	<0.001*	0.982	410
Sedative use disorder	122 (46%)	34 (63%)	77 (69%)	<0.001*[a]	0.043	<0.001*	0.571	434
Cocaine use disorder	248 (91%)	48 (96%)	100 (91%)	0.511	0.515	1	0.515	434
Alcohol use disorder	191 (71%)	44 (81%)	81 (76%)	0.194	0.444	0.492	0.596	430
Cannabis use disorder	215 (79%)	47 (92%)	88 (79%)	0.08	0.085	1	0.085	435
Opiates use disorder	204 (72%)	37 (69%)	86 (75%)	0.602	0.771	0.767	0.767	453
Number of SUDs ≥3	117 (41%)	34 (63%)	60 (53%)	0.003*[a]	0.013*	0.061	0.274	455
Gender (Women vs. Men)	51 (18%)	22 (41%)	30 (26%)	0.001*[a]	0.001*	0.087	0.087	455
Age	39 [32;45]	39 [29;45]	40 [32;46]	0.43	0.409	0.409	0.409	455
Homelessness ≥ 3 months	64 (26%)	13 (27%)	39 (38%)	0.076	1	0.105	0.398	398
Number of current medications	2 [1;4]	3 [1;4]	4 [2;4]	0.005*[a]	0.299	0.004*	0.217	455
Current antidepressant treatment	64 (22%)	19 (35%)	30 (26%)	0.121	0.192	0.468	0.468	455
Current number of psychotropic medications	1 [0;2]	2 [1;2]	2 [1;2]	<0.001*[a]	0.007*	0.001*	0.832	455
Current number of non-psychotropic medications	1 [0;2]	1 [0;2]	1 [0;2]	0.091	0.458	0.106	0.106	455
Protocol of origin (#2 vs. #1)	2 [1;2]	2 [1;2]	2 [1;2]	0.605	0.828	0.828	0.828	455

Supplementary Table 3: sample description and bivariate associations with serious suicide attempts (SA). Data are presented as median [interquartile range] or N (%). ***p* <0.05 after Bonferroni correction for three tests.** [a]test for trend significant at *p*<0.025 (Bonferroni correction for two rounds of tests applied for serious and recurrent SAs).

	No lifetime SA, N=287 (63%)	One lifetime SA, N=66 (15%)	≥2 lifetime SAs, N =102 (22%)	***p*-value (Chi^2 or Kruskal-Wallis)**				N
				**overall**	**No SA *vs.* only 1 SA**	**No SA *vs.* ≥2SAs**	**only 1 SA *vs.* ≥2 SAs**	
At least one, but not serious SA	0 (0%)	23 (35%)	31 (31%)	<0.001*[a]	<0.001	<0.001	0.614	454
At least one serious SA	0 (0%)	43 (65%)	70 (69%)					
Cigarettes per day, median (IQR)	15 [10;20]	20 [15;25]	20 [12;26]	<0.001*[a]	0.005	<0.001	0.626	410
Sedative use disorder	122 (46%)	38 (58%)	74 (73%)	<0.001*[a]	0.083	<0.001	0.083	434
Cocaine use disorder	248 (91%)	54 (87%)	93 (95%)	0.217	0.567	0.384	0.384	434
Alcohol use disorder	191 (71%)	49 (79%)	76 (78%)	0.24	0.37	0.37	0.98	430
Cannabis use disorder	215 (79%)	55 (86%)	80 (81%)	0.455	0.788	0.82	0.788	435
Opiates use disorder	204 (72%)	50 (76%)	73 (72%)	0.782	1	1	1	453
Number of SUDs ≥3	117 (41%)	32 (48%)	62 (61%)	0.002*[a]	0.314	0.002*	0.238	455
Gender (Women vs. Men)	51 (18%)	18 (27%)	34 (33%)	0.003*[a]	0.17	0.005*	0.51	455
Age (median, IQR)	39 [32;45]	40 [31;44]	40 [32;47]	0.558	0.516	0.516	0.516	455
Homelessness ≥ 3 months	64 (26%)	16 (28%)	36 (39%)	0.066	0.924	0.089	0.332	398
Number of current medications	2 [1;4]	4 [1;4]	3 [2;4]	0.008*[a]	0.045	0.026	0.912	455
Current antidepressant treatment	64 (22%)	20 (30%)	29 (28%)	0.253	0.399	0.399	0.931	455
Current number of psychotropic medications	1 [0;2]	2 [1;2]	2 [1;2]	<0.001*[a]	0.012*	<0.001*	0.559	455
Current number of non-psychotropic medications	1 [0;2]	1 [0;2]	1 [0;2]	0.478	0.588	0.588	0.588	455
Protocol of origin (#2 vs. #1)	2 [1;2]	2 [1;2]	2 [1;2]	0.498	0.816	0.714	0.752	455

Supplementary Table 4: sample description and bivariate associations with recurrent suicide attempts (SA). Data are presented as median [interquartile range] or N (%). ***p* <0.05 after Bonferroni correction for three tests.** [a]test for trend significant at *p* <0.025 (Bonferroni correction for two rounds of tests applied for serious and recurrent SAs).
